# Real-World Treatment Patterns of Antiviral Prophylaxis for Cytomegalovirus Among Adult Kidney Transplant Recipients: A Linked USRDS-Medicare Database Study

**DOI:** 10.3389/ti.2022.10528

**Published:** 2022-08-12

**Authors:** Amit D. Raval, Michael L. Ganz, Kathy Fraeman, Andrea L. Lorden, Shanmugapriya Saravanan, Yuexin Tang, Carlos A. Q. Santos

**Affiliations:** ^1^ Merck & Co., Inc., Kenilworth, NJ, United States; ^2^ Evidera, Bethesda, MD, United States; ^3^ Rush Medical College, Rush University, Chicago, IL, United States

**Keywords:** kidney transplantation, antiviral, cytomegalovirus, prophylaxis, pharmacoepidemiology

## Abstract

Limited data exist on cytomegalovirus (CMV) antiviral treatment patterns among kidney transplant recipients (KTRs). Using United States Renal Database System registry data and Medicare claims (1 January 2011–31 December 2017), we examined CMV antiviral use in 20,601 KTRs who received their first KT from 2011 to 2016. Three-quarters of KTRs started CMV prophylaxis (86.9% of high-, 83.6% of intermediate-, and 31.7% of low-risk KTRs). Median time to prophylaxis discontinuation was 121, 90, and 90 days for high-, intermediate-, and low-risk KTRs, respectively. Factors associated with receiving CMV prophylaxis were high-risk status, diabetes, receipt of a well-functioning kidney graft, greater time on dialysis before KT, panel reactive antibodies ≥80%, and use of antithymocyte globulin, alemtuzumab, and tacrolimus. KTRs were more likely to discontinue CMV prophylaxis if they developed leukopenia/neutropenia, had liver disease, or had a deceased donor. These findings suggest that adherence to the recommended duration of CMV-prophylaxis for high and intermediate-risk patients is suboptimal, and CMV prophylaxis is overused in low-risk patients.

## Graphical Abstract

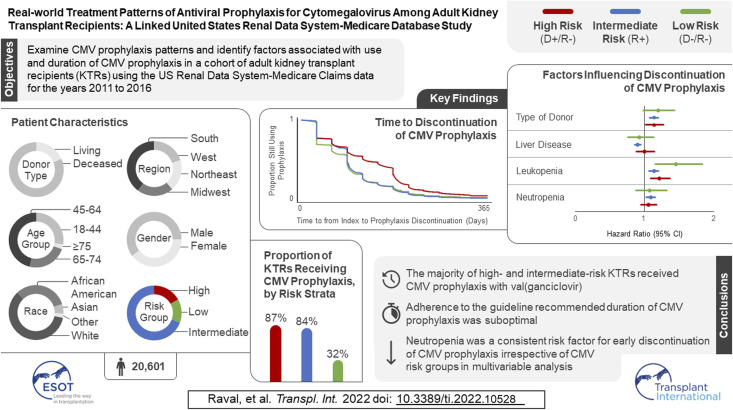

## Introduction

Cytomegalovirus (CMV) is the most common opportunistic infection in kidney transplant recipients (KTRs) (1, 2). In the absence of prevention, 20%–60% of KTRs develop CMV infection/disease. CMV infection and its manifestations increase the risk of rejection, graft loss, and mortality (1, 3). Previous research has shown that the use of CMV antiviral agents, including (val)ganciclovir, is associated with a reduced risk of CMV infection/disease (3–7). Prophylactic use of these antivirals not only lowers the risk of CMV infection/disease, but also mitigates the negative impact of CMV on graft and survival outcomes (3-7). However, currently available CMV antiviral agents may lead to adverse outcomes such as myelosuppression from (val)ganciclovir or nephrotoxicity from foscarnet, which may require modifications to antiviral or immunosuppressive therapy regimens that can also adversely affect graft and survival outcomes (3, 5, 7, 8).

CMV serostatus is a key determinant of CMV infection/disease risk. CMV seronegative KTRs (R–) who received a graft from a CMV seropositive donor (D+) are at the greatest risk for CMV infection/disease, followed by CMV seropositive (R+) KTRs regardless of donor serostatus (D±), who are at intermediate risk, and CMV seronegative KTRs who receive a graft from a CMV seronegative donor (D–/R–), who are at lowest risk of CMV infection/disease (9). CMV prevention is prioritized for high-risk KTRs, leading to a recommended 200 days of prophylaxis, while efficacy and safety are balanced for intermediate-risk KTRs, leading to a recommended duration of 100 days. CMV prophylaxis is not recommended for low-risk KTRs. The standard valganciclovir daily dose of 900 mg can be lowered to 450 mg to reduce the risk of myelosuppression if antiviral toxicities are a concern, but this strategy may be suboptimal (10).

While preemptive therapy can be substituted for prophylaxis if the KTR has the logistical support necessary for monitoring, a recent systematic review of post-transplant CMV preventive strategies for nearly 70,000 KTRs found that prophylaxis was the most common approach for high-risk transplants, preemptive therapy was the most common approach for intermediate-risk transplants, and ganciclovir or valganciclovir were identified as the most commonly used medications regardless of CMV risk (3). However, the majority of reported studies are limited to examining a single center, or are outdated due to updated guidelines supporting a longer duration of CMV prophylaxis consistent with results from the IMPACT clinical trial (11). Additionally, few studies have published CMV prophylaxis patterns among KTRs using large-scale national-level databases in the United States (US), leaving a gap in real-world evidence regarding the characteristics and determinants of CMV prophylaxis patterns among adult KTRs. Therefore, we conducted this study to determine patterns of CMV prophylaxis use and identify factors associated with use and duration of CMV prophylaxis.

## Materials and Methods

### Data Source

We used files from the US Renal Data System (USRDS) linked to Medicare claims between 1 January 2011 and 31 December 2017 (12). The USRDS is a national registry that collects treatment and outcomes data from individuals with chronic kidney disease and end-stage renal disease (ESRD) in the US. The USRDS-Medicare database is considered the most complete source of information on the use of healthcare services by KTRs in the US, because ESRD is a qualifying condition for Medicare coverage and the registry includes all individuals who require maintenance dialysis. The USRDS standard analysis files contain data on person-level clinical and demographic characteristics, kidney transplant (KT) information from the United Network of Organ Sharing (UNOS), and death. The standard USRDS files can be linked to Medicare Institutional (Part A), Physician/Supplier (Part B), and Prescription Drug (Part D) claims. This study was approved by the New England Institutional Review Board on 9 September 2020 (study number 1289813) and was conducted in accordance with the International Society for Pharmacoepidemiology Guidelines for Good Pharmacoepidemiology Practices, Revision 3, the principles of the Declaration of Helsinki, and all applicable federal, state, and local laws, rules, and regulations.

### Study Design and Sample

**FIGURE 1:**
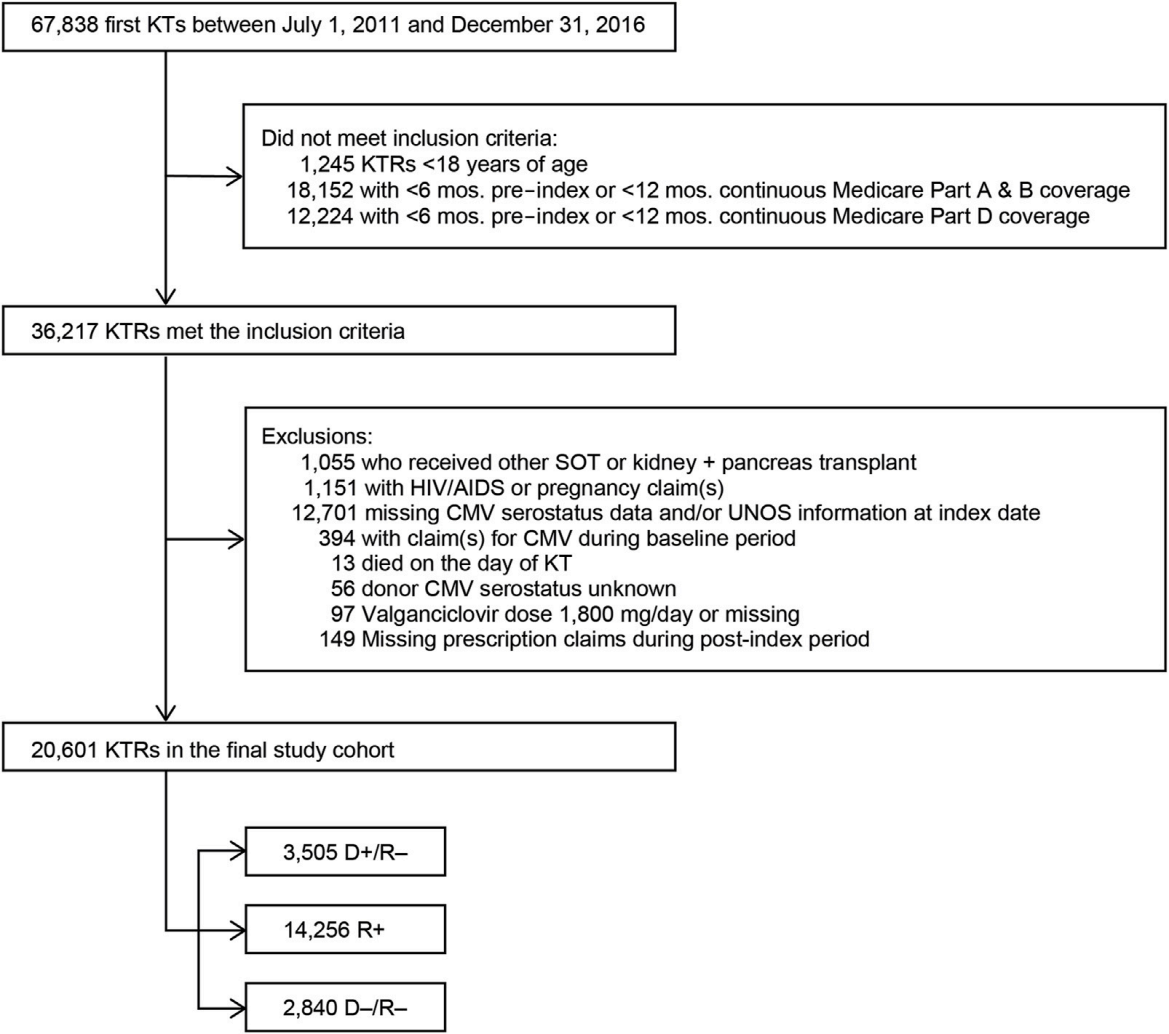
Study sample selection. Abbreviations: AIDS, acquired immunodeficiency syndrome; CMV, cytomegalovirus; D+, seropositive donor; D−, seronegative donor; HIV, human immunodeficiency virus; KT, kidney transplant; KTRs, kidney transplant recipients; mos., months; R+, seropositive recipient; R−, seronegative recipient; SOT, solid organ transplant.

We performed a retrospective, observational cohort analysis of individuals who were at least 18 years of age at the time of their first KT that occurred between 1 June 2011 and 31 December 2016. The claims-derived date of their first KT was used as the KTRs’ index dates. Included KTRs had to have at least one medical procedure claim for KT in the Medicare claims data within 15 days of the registry-based date of the KT; at least 6 months of continuous Medicare Parts A, B, and D coverage prior to their index date; and at least 12 months of continuous Medicare Parts A, B, and D coverage post-index date or continuous Medicare Part A, Part B, and Part D up to date of death if death occurred within 1 year of transplant. KT was identified by the International Classification of Diseases, Clinical Modification diagnosis codes 55.69 (ninth revision) and 0TY00Z0, 0TY00Z1, 0TY00Z2, 0TY10Z0, 0TY10Z1, and 0TY10Z2 (10th revision) in the Medicare Claims data. Once all KTRs who met inclusion criteria were identified, exclusion criteria were applied to identify our final cohort (Figure 1). Exclusion criteria included evidence of HIV/AIDS or pregnancy in claims data, missing CMV or UNOS information at index date, claim for CMV during the baseline period, died on day of KT, CMV serostatus missing, and valganciclovir dose missing or exceeded 1,800 mg/day.

### Definitions of CMV Prophylaxis and Duration

We defined CMV prophylactic therapy as use of ganciclovir or valganciclovir within 28 days after the KT index date. CMV antiviral therapies were identified in Part D Medicare claims using National Drug Codes for ganciclovir and valganciclovir, or Parts A or B Medicare claims using Healthcare Common Procedure Coding System (HCPCS) or Current Procedural Terminology (CPT) codes for administration of those agents. To calculate duration of CMV prophylaxis, we first identified the fill date and the days’ supply and then estimated the run-out date for each CMV antiviral prescription. We defined the index prescription as the first CMV antiviral medication within 28 days after the KT. Fill gaps were then calculated, where a fill gap was the difference between run-out date and the next fill date for the CMV prophylactic antiviral being used. Finally, we defined the last prophylaxis prescription by the first occurrence of a fill gap of ≥15 days after the index prescription. Duration was the difference between the last prophylaxis prescription run-out date and the index prescription fill date.

### Valganciclovir Daily Dose

We estimated the total daily dose (TDD) for each identified valganciclovir prescription by multiplying the strength of the prescription by the number of tablets dispensed, divided by the number of days supplied (e.g., 60 tablets of valganciclovir 450 mg dispensed for 30 days equals a TDD of 900 mg). Once calculated for each prescription, an average TDD for CMV prophylaxis was calculated for each KTR and used to classify KTRs to a valganciclovir daily dose category (450 mg, 900 mg, or other).

### Definitions of Leukopenia and Neutropenia

We created time-varying covariates to capture when KTRs developed leukopenia and/or neutropenia on or after their transplant dates. These time-varying covariates were defined using diagnosis codes present during hospitalizations and were equal to “no” until the date of their first relevant diagnosis code for each condition, after which point they were set equal to “yes.”

### Other KTR Characteristics

Demographic characteristics included age, gender, race (White, African American, other), ethnicity, and geographic region (Northeast, Midwest, South, West and other US territories). Clinical factors that may influence outcomes, which were used as covariates for adjusted analyses, included primary diagnosis leading to ESRD (diabetes of any type, hypertensive nephrosclerosis, polycystic kidney disease, focal glomerular sclerosis, other diseases), Charlson Comorbidity Index (CCI), other comorbid conditions (cardiovascular disease, chronic pulmonary disease, diabetes, liver disease, rheumatologic disease), donor type (deceased, living), cold ischemia time (≥24, <24 h), donor creatinine (≤1.5, >1.5 mg/dl), time on dialysis prior to KT (in months), human leukocyte antigens (HLA) A B match (≥3, <3), panel reactive antibodies (PRA; ≥80%, <80%), hepatitis C virus status, and Epstein Barr virus status. Two types of immunosuppressive therapies were considered. Induction agents included antithymocyte globulin (ATG), alemtuzumab, basiliximab, and other agents (daclizumab, muromonab-CD3, rituximab, and cyclophosphamide). Maintenance agents included mycophenolate mofetil (MMF), tacrolimus, azathioprine (AZA), everolimus, cyclosporine, prednisone and/or methylprednisolone, and other agents (sirolimus, leflunomide, belatacept, or any others identified as maintenance).

### Statistical Analysis

Summary statistics were used to describe the KTRs and their CMV prophylaxis patterns. Comparisons between groups were performed using the F-test from analysis of variance and the chi-square test for continuous and categorical variables, respectively. All analyses were stratified by CMV risk associated with the donor/recipient serostatus. Results for cells containing fewer than 11 KTRs have been suppressed (i.e., reported as “<11”) as required by the USRDS data use agreement. We generated Kaplan-Meier (KM) curves to visualize time to prophylaxis discontinuation and the log-rank test to assess differences between those curves. Multivariable logistic and Cox proportional hazard (PH) regression models were used to estimate the adjusted associations between KTRs’ demographic and clinical characteristics and the probabilities of starting and discontinuing, respectively, their CMV prophylaxis. Regression models were estimated for all KTRs while adjusting for risk group and separately by risk group, and results were reported as odds and hazard ratios for the logistic and Cox PH models, respectively, along with 95% confidence intervals and two-sided *p*-values. The logistic and PH Cox regression models included the same core set of covariates, which was selected based on the literature; the PH Cox models also included two time-varying covariates capturing post-KT occurrence of leukopenia and neutropenia. When variables were missing values, we applied the following imputation strategies. For continuous variables such as time on dialysis and time on the transplant waiting list, we replaced the missing values with the risk-group-specific means. For categorical variables such as cold ischemia time, PRA, and HLA A B match, we replaced the missing values with the risk group-specific modal value. Missing values for categorical cold ischemia time and donor creatinine level were imputed after imputing the source continuous variables.

## Results

### Baseline Characteristics

We identified 67,838 individuals who received their first KT from 2011 to 2016, of whom 20,601 satisfied all inclusion and exclusion criteria (Figure 1). Table 1 summarizes the characteristics of our sample. Most (69.2%) KTRs were at intermediate risk of CMV infection, while 17.0% and 13.8% were at high and low risk, respectively. KTRs were, on average, 53.2 years of age at their initial KT. Most KTRs were male (60.1%) and White (60.0%); one-third were African American. Diabetes (28.4%), hypertensive nephrosclerosis (27.8%), polycystic kidney disease (6.3%), focal glomerular sclerosis (5.6%), and systemic lupus erythematosus (3.6%) were the five most frequent primary diseases leading to ESRD. More than one-third (37.9%) of the KTRs had a CCI score ≥5, and nearly one-quarter of KTRs also had congestive heart failure (23.8%). KTRs spent, on average, 4.8 years on dialysis prior to their KT and 2.6 years on the transplant waiting list. Large proportions of KTRs received their kidney grafts from a deceased donor (81.5%) and were positive for Epstein-Barr virus (82.0%). Most donor kidneys experienced <24 h of cold ischemia time (81.6%) and were well-functioning (donor creatinine clearance ≤1.5 mg/dL). Approximately 22% had HLA A B donor-recipient match scores ≥3, and 9.2% of KTRs had PRA ≥80%. ATG was the most used induction immunosuppressive agent (54.7%), followed by basiliximab (22.2%) and alemtuzumab (16.5%). Almost all KTRs used prednisone and/or methylprednisolone (96.3%), MMF (96.3%), and tacrolimus (94.9%) as maintenance immunosuppressive agents. High-risk KTRs were more likely to have had PRA equal to zero, and high- and intermediate-risk KTRs were less likely to have had three or more HLA A B matches than other KTRs. Intermediaterisk KTRs were slightly older and more likely to be female, African American or Asian, Hispanic, reside in the South or West regions, have diabetes or hypertensive nephrosclerosis as the primary cause of ESRD, have a CCI score ≥5, and PRA ≥80% than KTRs in the other groups. Low-risk KTRs were more likely to reside in the Northeast or Midwest, and they were less likely to have had comorbid diabetes and to have used basiliximab as an induction immunosuppressive agent than other KTRs.

**TABLE 1:** Baseline demographic, clinical, and medication-related characteristics of adult KTRs.

Characteristic	Overall (*N* = 20,601)	High risk (D+/R–) (*N* = 3,505)	Intermediate risk (R+) (*N* = 14,256)	Low risk (D–/R–) (*N* = 2,840)	*p*-value^a^
Mean age in years (SD)	53.2 (14.0)	51.7 (14.6)	53.9 (13.6)	51.4 (15.0)	<0.01
Age category in years, *N* (%)					
18–44	5,670 (27.5%)	1,102 (31.4%)	3,601 (25.3%)	967 (34.0%)	<0.01
45–64	9,545 (46.3%)	1,538 (43.9%)	6,862 (48.1%)	1,145 (40.3%)
65–74	4,837 (23.5%)	779 (22.2%)	3,400 (23.8%)	658 (23.2%)	
≥75	549 (2.7%)	86 (2.5%)	393 (2.8%)	70 (2.5%)	
Gender, *N* (%)					
Male	12,383 (60.1%)	2,467 (70.4%)	7,951 (55.8%)	1,965 (69.2%)	<0.01
Female	8,218 (39.9%)	1,038 (29.6%)	6,305 (44.2%)	875 (30.8%)	
Race, *N* (%)					
White	12,366 (60.0%)	2,479 (70.7%)	7,757 (54.4%)	2,130 (75.0%)	<0.01
African American	6,600 (32.0%)	932 (26.6%)	5,029 (35.3%)	639 (22.5%)	
Asian	1,147 (5.6%)	50 (1.4%)	1,060 (7.4%)	37 (1.3%)	
Other^b^					
Hispanic ethnicity, *N* (%)					
Yes	4,346 (21.1%)	435 (12.4%)	3,642 (25.5%)	269 (9.5%)	<0.01
No	16,093 (78.1%)	3,037 (86.6%)	10,504 (73.7%)	2,552 (89.9%)	
Unknown	162 (0.8%)	33 (0.9%)	110 (0.8%)	19 (0.7%)	
Geographic region, *N* (%)					
Northeast	3,830 (18.6%)	720 (20.5%)	2,406 (16.9%)	704 (24.8%)	<0.01
Midwest	4,424 (21.5%)	815 (23.3%)	2,822 (19.8%)	787 (27.7%)	
South	8,156 (39.6%)	1,377 (39.3%)	5,869 (41.2%)	910 (32.0%)	
West	4,137 (20.1%)	589 (16.8%)	3,123 (21.9%)	425 (15.0%)	
Other US territories	54 (0.3%)	<11	36 (0.3%)	14 (0.5%)	
Primary diagnosis leading to ESRD, *N* (%)					
Diabetes mellitus, Type 2	5,843 (28.4%)	873 (24.9%)	4,343 (30.5%)	627 (22.1%)	<0.01
Hypertensive nephrosclerosis	5,724 (27.8%)	863 (24.6%)	4,130 (29.0%)	731 (25.7%)	
Polycystic kidney disease	1,289 (6.3%)	255 (7.3%)	826 (5.8%)	208 (7.3%)	
Focal glomerular sclerosis	1,157 (5.6%)	221 (6.3%)	761 (5.3%)	175 (6.2%)
Systemic lupus erythematosus	751 (3.6%)	108 (3.1%)	561 (3.9%)	82 (2.9%)	
Diabetes mellitus - Type I	720 (3.5%)	146 (4.2%)	427 (3.0%)	147 (5.2%)	
IGA nephropathy	669 (3.2%)	125 (3.6%)	421 (3.0%)	123 (4.3%)	
Chronic glomerulonephritis unspecified	502 (2.4%)	88 (2.5%)	343 (2.4%)	71 (2.5%)	
Malignant hypertension	250 (1.2%)	46 (1.3%)	174 (1.2%)	30 (1.1%)	
Membranous glomerulonephritis	199 (1.0%)	47 (1.3%)	126 (0.9%)	26 (0.9%)	
Other Disease	3,497 (17.0%)	733 (20.9%)	2,144 (15.0%)	620 (21.8%)	
Charlson Comorbidity Index, *N* (%)					
0	0 (0.0%)	0 (0.0%)	0 (0.0%)	0 (0.0%)	<0.01
1–2	4,683 (22.7%)	839 (23.9%)	3,114 (21.8%)	730 (25.7%)	
3–4	8,110 (39.4%)	1,438 (41.0%)	5,525 (38.8%)	1,147 (40.4%)	
≥5	7,808 (37.9%)	1,228 (35.0%)	5,617 (39.4%)	963 (33.9%)	
Comorbid health conditions, *N* (%)					
Congestive heart failure	4,912 (23.8%)	782 (22.3%)	3,483 (24.4%)	647 (22.8%)	0.01
Diabetes	9,091 (44.1%)	1,441 (41.1%)	6,565 (46.1%)	1,085 (38.2%)	<0.01
Diabetes without chronic complication	3,948 (19.2%)	635 (18.1%)	2,802 (19.7%)	511 (18.0%)	0.03
Diabetes with chronic complication	8,586 (41.7%)	1,358 (38.7%)	6,220 (43.6%)	1,008 (35.5%)	<0.01
Chronic pulmonary disease	3,345 (16.2%)	587 (16.7%)	2,288 (16.0%)	470 (16.5%)	0.54
Peripheral vascular disease	5,025 (24.4%)	849 (24.2%)	3,575 (25.1%)	601 (21.2%)	<0.01
Rheumatologic disease	1,389 (6.7%)	208 (5.9%)	1,016 (7.1%)	165 (5.8%)	<0.01
Mild to moderate liver disease	3,016 (14.6%)	482 (13.8%)	2,147 (15.1%)	387 (13.6%)	0.04
Sever liver disease	91 (0.4%)	<11	75 (0.5%)	<11	0.02
Myocardial infarction	1,843 (8.9%)	307 (8.8%)	1,275 (8.9%)	261 (9.2%)	0.84
Dementia	164 (0.8%)	38 (1.1%)	96 (0.7%)	30 (1.1%)	0.01
Mean time on dialysis prior to KT (SD), years	4.8 (3.2)	4.6 (3.1)	4.9 (3.3)	4.1 (3.1)	<0.01
Mean wait time (SD), years	2.6 (2.1)	2.5 (2.1)	2.6 (2.2)	2.2 (1.9)	<0.01
PRA, *N* (%)					
0%	13,565 (65.8%)	2,498 (71.3%)	9,066 (63.6%)	2,001 (70.5%)	<0.01
1%–19%	1,791 (8.7%)	308 (8.8%)	1,240 (8.7%)	243 (8.6%)	
20%–79%	3,100 (15.0%)	464 (13.2%)	2,255 (15.8%)	381 (13.4%)	
80%–100%	1,898 (9.2%)	196 (5.6%)	1,555 (10.9%)	147 (5.2%)	
Missing	247 (1.2%)	39 (1.1%)	140 (1.0%)	68 (2.4%)	
HLA A B donor-recipient match, *N* (%)					
0	4,338 (21.1%)	701 (20.0%)	3,086 (21.6%)	551 (19.4%)	<0.01
1	6,872 (33.4%)	1,217 (34.7%)	4,778 (33.5%)	877 (30.9%)	
2	4,553 (22.1%)	777 (22.2%)	3,127 (21.9%)	649 (22.9%)	
≥3	4,601 (22.3%)	766 (21.9%)	3,104 (21.8%)	731 (25.7%)	
Missing	237 (1.2%)	44 (1.3%)	161 (1.1%)	32 (1.1%)	
Hepatitis C seropositive, *N* (%)	849 (4.1%)	108 (3.1%)	648 (4.5%)	93 (3.3%)	<0.01
Epstein-Barr virus antibody positive, *N* (%)	16,887 (82.0%)	2,737 (78.1%)	11,864 (83.2%)	2,286 (80.5%)	<0.01
Calendar year of transplant, *N* (%)					
2011	1,857 (9.0%)	338 (9.6%)	1,287 (9.0%)	232 (8.2%)	<0.01
2012	3,613 (17.5%)	607 (17.3%)	2,523 (17.7%)	483 (17.0%)	
2013	3,552 (17.2%)	598 (17.1%)	2,515 (17.6%)	439 (15.5%)	
2014	3,516 (17.1%)	609 (17.4%)	2,441 (17.1%)	466 (16.4%)	
2015	3,950 (19.2%)	659 (18.8%)	2,679 (18.8%)	612 (21.5%)	
2016	4,113 (20.0%)	694 (19.8%)	2,811 (19.7%)	608 (21.4%)	
Used immunosuppressive agents, *N* (%)	20,376 (98.9%)	3,466 (98.9%)	14,092 (98.8%)	2,818 (99.2%)	0.21
Induction immunosuppressive therapy, *N* (%)					
ATG	11,148 (54.7%)	1,801 (52.0%)	7,808 (55.4%)	1,539 (54.6%)	<0.01
Basiliximab	4,518 (22.2%)	805 (23.2%)	3,114 (22.1%)	599 (21.3%)	0.16
Alemtuzumab	3,369 (16.5%)	600 (17.3%)	2,316 (16.4%)	453 (16.1%)	0.36
Rituximab	142 (0.7%)	12 (0.3%)	117 (0.8%)	13 (0.5%)	<0.01
Muromonab-CD3	20 (0.10%)	<11	<11	<11	0.02
Daclizumab	<11	0 (0.0%)	<11	0 (0.0%)	NA
Cyclophosphamide					
Maintenance immunosuppressive therapy, *N* (%)					
Prednisone or methylprednisolone	19,623 (96.3%)	3,320 (95.8%)	13,595 (96.5%)	2,708 (96.1%)	0.13
MMF	19,624 (96.3%)	3,328 (96.0%)	13,613 (96.6%)	2,683 (95.2%)	<0.01
Tacrolimus	19,327 (94.9%)	3,272 (94.4%)	13,383 (95.0%)	2,672 (94.8%)	0.40
Belatacept	530 (2.6%)	89 (2.6%)	381 (2.7%)	60 (2.1%)	0.21
Cyclosporine	399 (2.0%)	70 (2.0%)	275 (2.0%)	54 (1.9%)	0.95
Sirolimus	239 (1.2%)	45 (1.3%)	144 (1.0%)	50 (1.8%)	<0.01
Everolimus	207 (1.0%)	44 (1.3%)	125 (0.9%)	38 (1.3%)	0.02
Leflunomide	11 (0.05%)	<11	<11	<11	0.72
AZA	65 (0.3%)	12 (0.3%)	42 (0.3%)	11 (0.4%)	0.70
Other	338 (1.7%)	53 (1.5%)	248 (1.8%)	37 (1.3%)	0.19
Donor type, *N* (%)					
Deceased	16,789 (81.5%)	2,907 (82.9%)	11,866 (83.2%)	2,016 (71.0%)	<0.01
Living	3,812 (18.5%)	598 (17.1%)	2,390 (16.8%)	824 (29.0%)	
Mean cold ischemia time in hours (SD)	14.9 (10.0)	14.7 (9.6)	15.4 (10.0)	12.9 (9.9)	<0.01
Cold ischemia time in hours category, *N* (%)					
<24 h	16,807 (81.6%)	2,896 (82.6%)	11,514 (80.8%)	2,397 (84.4%)	<0.01
≥24 h	3,443 (16.7%)	551 (15.7%)	2,537 (17.8%)	355 (12.5%)	
Missing	351 (1.7%)	58 (1.7%)	205 (1.4%)	88 (3.1%)	
Mean donor creatinine in mg/dL (SD)	1.1 (1.0)	1.1 (1.1)	1.1 (0.9)	1.1 (0.9)	0.03
Donor creatinine in mg/dL category, *N* (%)					
≤1.5 mg/dL	17,187 (83.4%)	2,935 (83.7%)	11,817 (82.9%)	2,435 (85.7%)	<0.01
>1.5 mg/dL	3,399 (16.5%)	568 (16.2%)	2,429 (17.0%)	402 (14.2%)	
Missing	15 (0.07%)	<11	<11	<11	

Abbreviations: ATG, antithymocyte globulin; AZA, azathioprine; D, donor; D+, seropositive donor; D–, seronegative donor; ESRD, end-stage renal disease; HLA, human leukocyte antigen; IGA, immunoglobulin A; KT, kidney transplant; MMF, mycophenolate mofetil; NA, not applicable; PRA, panel-reactive antibody; R, recipient; R+, seropositive recipient; R–, seronegative recipient; SD, standard deviation; US, United States.

^a^ *p*-values are compared across patients by donor/recipient serostatus group using t-tests or analysis of variance (ANOVA) for continuous variables or chi-square tests for categorical variables.

^b^ Other includes American Indian, Alaska Native, Native Hawaiian, Pacific Islander, multiracial, other, and unknown.

### Use and Factors Associated With the Use of CMV Antiviral Prophylaxis

Table 2 displays, and compares across risk groups, the CMV prophylaxis characteristics of KTRs who started CMV prophylaxis. Slightly over three-quarters (77.0%) of KTRs started CMV prophylaxis (86.9% of high-, 83.6% of intermediate-, and 31.7% of low-risk KTRs). Overall, 59.7% and 32.5% of KTRs who started CMV prophylaxis used valganciclovir 450 mg and 900 mg, respectively, while 7.8% used other doses of valganciclovir; no patients used ganciclovir. Overall, KTRs who started prophylaxis did so, on average, 4.2 days after receiving their KTs; time to starting prophylaxis did not vary substantially across risk groups (4.1–4.5 days).

**TABLE 2:** Characteristics of CMV prophylaxis among adults undergoing first kidney transplant by serostatus.

Prophylaxis Information	Overall (*N* = 20,601)	High risk (D+/R–) (*N* = 3,505)	Intermediate risk (R+) (*N* = 14,256)	Low risk (D–/R–) (*N* = 2,840)	*p*-value
All prophylaxis agents					
CMV prophylaxis					
No prophylaxis	4,742 (23.0%)	459 (13.1%)	2,343 (16.4%)	1,940 (68.3%)	<0.01
Prophylaxis	15,859 (77.0%)	3,046 (86.9%)	11,913 (83.6%)	900 (31.7%)	
Type of prophylaxis, *N* (%)					
Valganciclovir	15,859 (100.0%)	3,046 (100.0%)	11,913 (100.0%)	900 (100.0%)	NA
Index dose 450 mg	9,462 (59.7%)	1,450 (47.6%)	7,518 (63.1%)	494 (54.9%)	<0.01
Index dose 900 mg	5,153 (32.5%)	1,371 (45.0%)	3,461 (29.1%)	321 (35.7%)	
Other index dose	1,244 (7.8%)	225 (7.4%)	934 (7.8%)	85 (9.4%)	
Ganciclovir					
Mean time to initiate any CMV prophylaxis in days (SD)	4.2 (4.4)	4.5 (4.7)	4.1 (4.3)	4.4 (4.9)	<0.01
Mean duration of CMV prophylaxis in days (SD)	107.5 (74.4)	134.1 (90.5)	101.5 (68.1)	97.6 (74.1)	<0.01
Duration of CMV prophylaxis, *N* (%)					
≥72 days	10,297 (64.9%)	2,034 (66.8%)	7,752 (65.1%)	511 (56.8%)	<0.01
≥90 days	9,912 (62.5%)	1,986 (65.2%)	7,433 (62.4%)	493 (54.8%)	<0.01
≥100 days	6,359 (40.1%)	1,700 (55.8%)	4,352 (36.5%)	307 (34.1%)	<0.01
≥180 days	3,201 (20.2%)	1,187 (39.0%)	1,868 (15.7%)	146 (16.2%)	<0.01
≥200 days	1,733 (10.9%)	583 (19.1%)	1,062 (8.9%)	88 (9.8%)	<0.01
Valganciclovir 450 mg					
Mean time to initiate valganciclovir 450 mg prophylaxis in days (SD)	4.0 (4.2)	4.4 (4.8)	3.9 (4.1)	4.4 (4.8)	<0.01
Mean duration of valganciclovir 450 mg prophylaxis in days (SD)	115.2 (75.4)	151.0 (91.0)	108.7 (69.7)	109.6 (79.8)	<0.01
Duration of valganciclovir 450 mg prophylaxis, *N* (%)					
≥72 days	6,786 (71.7%)	1,093 (75.4%)	5,376 (71.5%)	317 (64.2%)	<0.01
≥90 days	6,587 (69.6%)	1,072 (73.9%)	5,206 (69.2%)	309 (62.6%)	<0.01
≥100 days	4,123 (43.6%)	928 (64.0%)	2,998 (39.9%)	197 (39.9%)	<0.01
≥180 days	2,151 (22.7%)	691 (47.7%)	1,357 (18.1%)	103 (20.9%)	<0.01
≥200 days	1,151 (12.2%)	340 (23.4%)	749 (10.0%)	62 (12.6%)	<0.01
Valganciclovir 900 mg					
Mean time to initiate valganciclovir 900 mg prophylaxis in days (SD)	3.9 (4.3)	4.2 (4.5)	3.8 (4.2)	3.7 (4.4)	0.02
Mean duration of valganciclovir 900 mg prophylaxis in days (SD)	87.7 (67.8)	111.8 (84.5)	79.6 (58.5)	72.3 (55.6)	<0.01
Duration of valganciclovir 900 mg prophylaxis, *N* (%)					
≥72 days	2,513 (48.8%)	760 (55.4%)	1,622 (46.9%)	131 (40.8%)	<0.01
≥90 days	2,413 (46.8%)	739 (53.9%)	1,544 (44.6%)	130 (40.5%)	<0.01
≥100 days	1,484 (28.8%)	614 (44.8%)	807 (23.3%)	63 (19.6%)	<0.01
≥180 days	716 (13.9%)	392 (28.6%)	305 (8.8%)	19 (5.9%)	<0.01
≥200 days	361 (7.0%)	174 (12.7%)	177 (5.1%)	<11	<0.01
Valganciclovir other dose					
Mean time to initiate valganciclovir other dose in days (SD)	6.6 (5.3)	6.9 (5.3)	6.5 (5.2)	6.8 (6.4)	0.57
Mean duration of valganciclovir other dose prophylaxis in days (SD)	131.5 (74.9)	160.9 (92.0)	125.1 (68.4)	123.7 (74.5)	<0.01
Duration of valganciclovir other dose prophylaxis, *N* (%)					
≥72 days	998 (80.2%)	181 (80.4%)	754 (80.7%)	63 (74.1%)	0.34
≥90 days	912 (73.3%)	175 (77.8%)	683 (73.1%)	54 (63.5%)	0.04
≥100 days	752 (60.5%)	158 (70.2%)	547 (58.6%)	47 (55.3%)	<0.01
≥180 days	334 (26.8%)	104 (46.2%)	206 (22.1%)	24 (28.2%)	<0.01
≥200 days	221 (17.8%)	69 (30.7%)	136 (14.6%)	16 (18.8%)	<0.01

Abbreviations: CMV, cytomegalovirus; D, donor; D+, seropositive donor; D–, seronegative donor; R, recipient; R+, seropositive recipient; R–, seronegative recipient; SD, standard deviation.

Table 3 displays the results of the logistic regression models for use of CMV prophylaxis (descriptive statistics stratified by CMV prophylaxis status within risk group are available in Supplementary Table 1). In general, CMV risk status was the factor most strongly associated with the use of CMV prophylaxis. KTRs who were younger, female, African American or of other races, resided in the Northeast, as well as those whose donor creatinine levels were >1.5 mg/dL, who spent more time on dialysis prior to KT, had PRA ≥80%, and who used ATG, and alemtuzumab were more likely to receive CMV prophylaxis (all and intermediate-risk KTRs). KTRs whose kidney graft experienced cold ischemia time <24 h, used basiliximab, AZA, everolimus, or cyclosporine, or prednisone and/or methylprednisolone were less likely to receive CMV prophylaxis (all and intermediate-risk KTRs). Additionally, high-risk KTRs who had PRA ≥80% were more likely to receive CMV prophylaxis; whereas those with comorbid diabetes, and who used AZA, everolimus, or cyclosporine, MMF or other maintenance immunosuppressive agents were less likely to receive CMV prophylaxis. Low-risk KTRs who were female, resided in the South, and used ATG and alemtuzumab or other immunosuppression as induction immunosuppressive agents were more likely to receive CMV prophylaxis.

**TABLE 3:** Logistic regression for probability of starting CMV prophylaxis among adults undergoing a first kidney transplant.

Predictors	Overall		High risk (D+/R–)		Intermediate risk (R+)		Low risk (D–/R–)	
	OR (95% CI)	*p*-value	OR (95% CI)	*p*-value	OR (95% CI)	*p*-value	OR (95% CI)	*p*-value
CMV serostatus (vs. D–/R–)								
D+/R–	17.16 (15.04–19.59)	<0.01						
R+	11.49 (10.42–12.67)	<0.01						
Age 18–64 years (vs. age ≥65)	1.61 (1.48–1.76)	<0.01	1.91 (1.52–2.40)	<0.01	1.64 (1.48–1.82)	<0.01	1.36 (1.10–1.69)	<0.01
Female gender (vs. male)	1.15 (1.06–1.25)	<0.01	1.01 (0.80–1.28)	0.90	1.19 (1.08–1.32)	<0.01	1.22 (1.00–1.47)	0.05
Race (vs. White)								
African American	1.15 (1.05–1.26)	<0.01	1.36 (1.04–1.78)	0.08	1.11 (0.99–1.24)	0.08	1.12 (0.90–1.39)	0.29
Other^a^	1.55 (1.32–1.82)	<0.01	2.25 (0.97–5.25)	0.06	1.42 (1.20–1.69)	<0.01	1.95 (1.18–3.22)	<0.01
Region (vs. Northeast)								
Midwest	0.55 (0.49–0.62)	<0.01	0.71 (0.53–0.96)	0.03	0.41 (0.35–0.48)	<0.01	0.66 (0.52–0.84)	<0.01
South	0.85 (0.76–0.95)	<0.01	0.85 (0.64–1.14)	0.28	0.60 (0.51–0.70)	<0.01	1.63 (1.30–2.03)	<0.01
West and Other US territories	0.85 (0.75–0.97)	0.02	1.10 (0.77–1.56)	0.61	0.69 (0.58–0.82)	<0.01	0.85 (0.65–1.13)	0.27
Primary disease leading to ESRD (vs. diabetes of any type)								
Hypertensive nephrosclerosis	1.18 (1.03–1.34)	0.02	0.99 (0.70–1.39)	0.94	1.32 (1.12–1.56)	<0.01	0.98 (0.72–1.34)	0.92
Polycystic kidney disease	1.06 (0.88–1.28)	0.54	0.95 (0.59–1.52)	0.82	1.17 (0.92–1.49)	0.19	0.86 (0.57–1.31)	0.49
Focal glomerular sclerosis	1.15 (0.95–1.40)	0.16	1.34 (0.76–2.37)	0.30	1.26 (0.98–1.62)	0.07	0.82 (0.53–1.26)	0.37
Other	1.09 (0.95–1.25)	0.23	0.90 (0.64–1.27)	0.56	1.27 (1.07–1.51)	<0.01	0.81 (0.59–1.10)	0.18
CCI ≥5 (vs. <5)	1.10 (0.98–1.23)	0.12	1.04 (0.76–1.41)	0.82	1.13 (0.98–1.31)	0.10	1.03 (0.79–1.33)	0.82
Comorbid health conditions								
Cardiovascular disease	0.94 (0.86–1.03)	0.18	0.89 (0.70–1.14)	0.35	0.90 (0.81–1.01)	0.07	1.11 (0.91–1.35)	0.32
Chronic pulmonary disease	0.91 (0.82–1.01)	0.07	1.02 (0.77–1.35)	0.89	0.83 (0.73–0.95)	<0.01	1.04 (0.83–1.31)	0.72
Diabetes	1.12 (0.99–1.27)	0.08	0.75 (0.54–1.04)	0.09	1.27 (1.08–1.50)	<0.01	1.05 (0.79–1.40)	0.73
Liver disease	1.05 (0.94–1.18)	0.37	1.09 (0.80–1.49)	0.59	1.04 (0.91–1.20)	0.54	1.02 (0.80–1.31)	0.85
Rheumatologic disease	0.88 (0.75–1.03)	0.10	0.92 (0.58–1.45)	0.71	0.87 (0.72–1.05)	0.15	0.88 (0.60–1.27)	0.48
Donor type deceased (vs. living)	0.91 (0.81–1.01)	0.07	0.82 (0.61–1.11)	0.20	0.93 (0.81–1.06)	0.28	0.93 (0.75–1.16)	0.54
Cold ischemia time <24 h (vs. ≥24 h)	0.95 (0.86–1.06)	0.37	1.18 (0.89–1.55)	0.26	0.92 (0.81–1.05)	0.23	0.89 (0.69–1.15)	0.38
Donor creatinine >1.5 mg/dL (vs. ≤1.5 mg/dL)	1.21 (1.09–1.36)	<0.01	1.22 (0.91–1.64)	0.19	1.21 (1.06–1.40)	<0.01	1.13 (0.88–1.45)	0.34
Time on dialysis prior to KT in years	1.02 (1.01–1.04)	<0.01	1.01 (0.97–1.06)	0.53	1.02 (1.01–1.04)	0.01	1.02 (0.99–1.06)	0.16
Wait time in years	0.99 (0.97–1.02)	0.62	1.00 (0.95–1.06)	0.98	0.99 (0.97–1.02)	0.47	1.01 (0.96–1.06)	0.77
PRAs ≥80% (vs. <80%)	1.36 (1.17–1.59)	<0.01	1.73 (1.00–2.98)	0.05	1.29 (1.08–1.55)	<0.01	1.33 (0.92–1.94)	0.13
HLA A B donor-recipient match ≥3 (vs. <3)	0.95 (0.87–1.04)	0.28	1.07 (0.83–1.37)	0.60	0.91 (0.81–1.02)	0.11	0.98 (0.80–1.20)	0.86
Calendar year of KT 2011–2013 (vs. 2014–2016)	1.12 (1.04–1.20)	<0.01	1.10 (0.90–1.35)	0.37	1.28 (1.17–1.41)	<0.01	0.73 (0.62–0.87)	<0.01
Induction immunosuppressive therapy^b^ (vs. absence of therapy)								
ATG	1.77 (1.59–1.97)	<0.01	1.18 (0.89–1.57)	0.26	2.04 (1.79–2.32)	<0.01	1.62 (1.26–2.07)	<0.01
Alemtuzumab	1.55 (1.36–1.78)	<0.01	1.06 (0.74–1.52)	0.73	1.67 (1.41–1.97)	<0.01	1.60 (1.19–2.16)	<0.01
Basiliximab	0.79 (0.70–0.88)	<0.01	0.97 (0.71–1.32)	0.84	0.71 (0.62–0.81)	<0.01	1.02 (0.78–1.35)	0.87
Other immunosuppression	1.69 (1.03–2.77)	0.04	0.69 (0.19–2.49)	0.57	1.39 (0.78–2.46)	0.27	4.48 (1.67–12.05)	<0.01
Maintenance immunosuppressive therapy^c^ (vs. absence of therapy)								
MMF	1.07 (0.88–1.30)	0.5	0.73 (0.42–1.25)	0.25	1.39 (1.10–1.76)	<0.01	0.81 (0.53–1.25)	0.34
Tacrolimus	1.16 (0.96–1.40)	0.11	0.96 (0.59–1.57)	0.87	1.50 (1.20–1.88)	<0.01	0.56 (0.36–0.87)	0.01
AZA, everolimus, and/or cyclosporine	0.37 (0.29–0.46)	<0.01	0.77 (0.41–1.47)	0.43	0.34 (0.26–0.44)	<0.01	0.56 (0.30–1.04)	0.07
Other immunosuppression	1.14 (0.95–1.37)	0.15	0.74 (0.46–1.17)	0.20	1.53 (1.22–1.92)	<0.01	0.71 (0.46–1.09)	0.12
Prednisone or methylprednisolone	0.54 (0.44–0.66)	<0.01	1.28 (0.82–1.98)	0.28	0.38 (0.28–0.51)	<0.01	0.52 (0.35–0.76)	<0.01

Abbreviations: ATG, antithymocyte globulin; AZA, azathioprine; CCI, Charlson comorbidity index; CI, confidence interval; CMV, cytomegalovirus; D, donor; D+, seropositive donor; D–, seronegative donor; ESRD, end-stage renal disease; HLA, human leukocyte antigen; KT, kidney transplant; MMF, mycophenolate mofetil; OR, odds ratio; PRA, panel-reactive antibody; R, recipient; R+, seropositive recipient; R–, seronegative recipient; US, United States.

^a^ Other includes Asian, American Indian, Alaska Native, Native Hawaiian, Pacific Islander, multiracial, other, and unknown.

^b^ Other immunosuppression therapies included daclizumab, muromonab-CD3, rituximab, and cyclophosphamide.

^c^ Other immunosuppression maintenance therapies included sirolimus, leflunomide, belatacept, or any other.

### Duration of Prophylaxis and Factors Associated With Risk of CMV Prophylaxis Discontinuation

**FIGURE 2:**
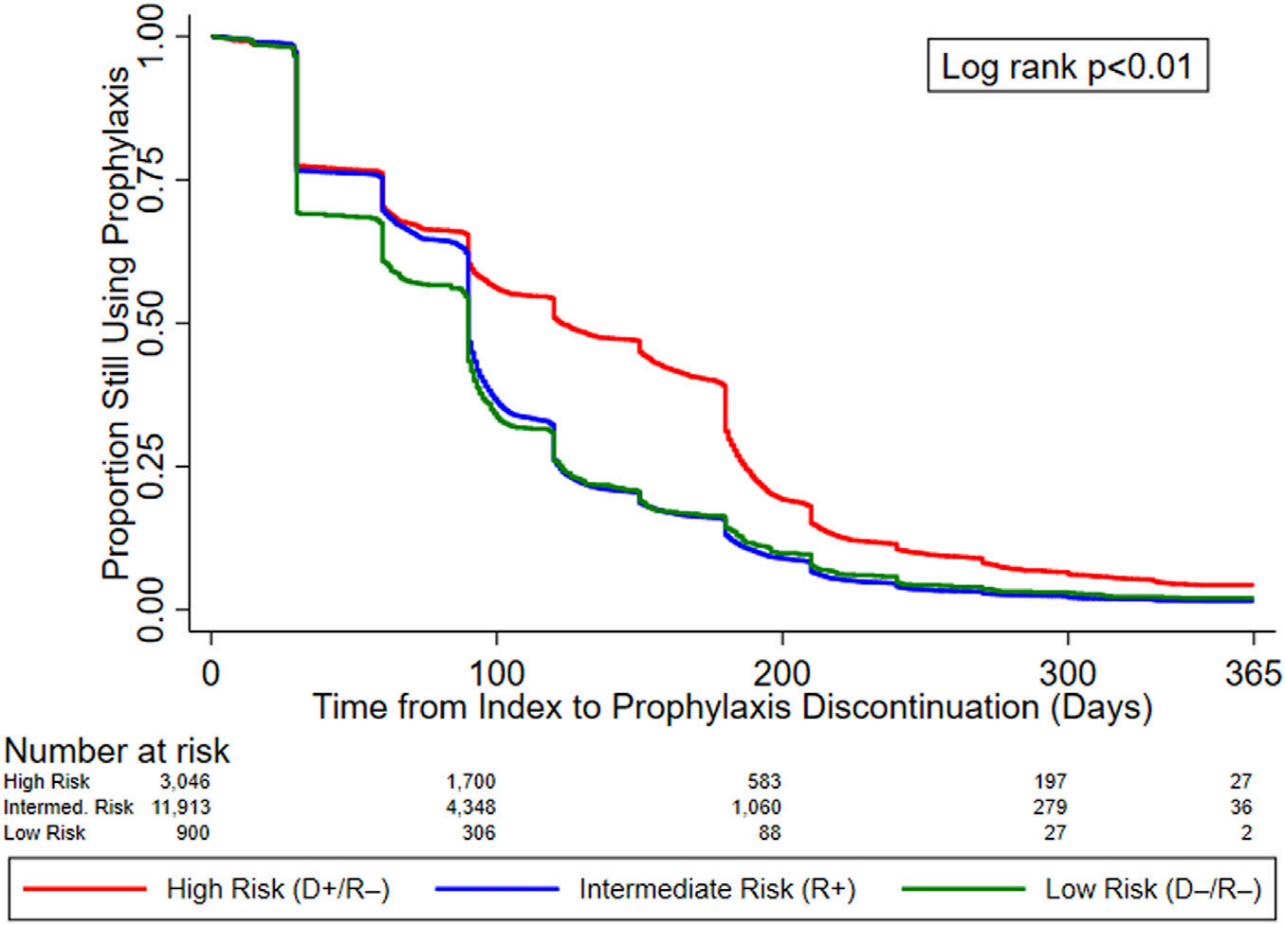
KM curves for time to prophylaxis discontinuation, by serostatus (CMV Risk Group). Abbreviations: D+, seropositive donor; D−, seronegative donor; R+, seropositive recipient; R−, seronegative recipient.

Figure 2 displays the KM curves for time to prophylaxis discontinuation. The median time to prophylaxis discontinuation (i.e., prophylaxis duration), derived from the KM curves, for the high-risk group of KTRs was longer (121 days) than for intermediate- (90 days) and low-risk (90 days) KTRs. Regardless of type of antiviral agent used, 10.9% of KTRs who used CMV prophylaxis did so for ≥200 days (23.4% and 12.7% of high-risk KTRs who used valganciclovir 450 mg and 900 mg, respectively, did so for ≥200 days) and more than half (55.8%) of high-risk KTRs used CMV prophylaxis for ≥100 days (64.0% and 44.8% of high-risk KTRs who used valganciclovir 450 mg and 900 mg, respectively, did so for ≥100 days). Over one-third (36.5%) of intermediate-risk KTRs used CMV prophylaxis for ≥100 days (39.4% and 23.3% of intermediate-risk KTRs who used valganciclovir 450 mg and 900 mg, respectively, did so for ≥100 days).

Table 4 displays the results of the PH Cox regression models for time to CMV prophylaxis discontinuation. We found that, regardless of risk group, KTRs who resided in the South and who developed leukopenia were more likely to discontinue CMV prophylaxis; all KTRs, as well as intermediate-risk group KTRs who developed neutropenia were also more likely to discontinue. Additionally, overall and intermediate-risk KTRs with comorbid liver disease, who experienced a longer wait time, lived in the Midwest, or received MMF or tacrolimus were more likely to discontinue CMV prophylaxis. Among the overall, high-, and intermediate-risk KTRs, those who were younger, received kidney grafts from deceased donors, or lived in the West or other US territories were more likely to discontinue prophylaxis. Finally, overall, intermediate-, and low-risk KTRs who identified as African American were more likely to discontinue CMV prophylaxis, as were overall and intermediate-risk KTRs of other races.

**TABLE 4:** Cox proportional hazard regression for time to CMV prophylaxis discontinuation among adults undergoing a first kidney transplant.

Predictors	Overall		High risk (D+/R–)		Intermediate risk (R+)		Low risk (D–/R–)	
	HR (95% CI)	*p*-value	HR (95% CI)	*p*-value	HR (95% CI)	*p*-value	HR (95% CI)	*p*-value
CMV serostatus (vs. D–/R–)								
D+/R–	0.60 (0.56–0.65)	<0.01						
R+	0.96 (0.89–1.03)	0.24						
Time-varying covariates (vs. no condition)								
Neutropenia	1.08 (1.03–1.14)	<0.01	1.06 (0.95–1.18)	0.28	1.09 (1.03–1.16)	<0.01	1.08 (0.88–1.33)	0.47
Leukopenia	1.17 (1.11–1.24)	<0.01	1.22 (1.09–1.37)	<0.01	1.14 (1.07–1.21)	<0.01	1.46 (1.16–1.85)	<0.01
Age 18–64 (vs. age ≥65)	0.84 (0.80–0.87)	<0.01	0.77 (0.70–0.84)	<0.01	0.84 (0.81–0.88)	<0.01	0.86 (0.71–1.03)	0.11
Female (vs. Male)	0.97 (0.93–1.00)	0.05	0.95 (0.87–1.04)	0.25	0.97 (0.93–1.01)	0.11	0.98 (0.84–1.15)	0.83
Race (vs. White)								
African American	1.08 (1.04–1.12)	<0.01	1.02 (0.93–1.12)	0.62	1.07 (1.03–1.12)	<0.01	1.26 (1.06–1.49)	<0.01
Other^a^	0.91 (0.86–0.97)	<0.01	1.17 (0.94–1.46)	0.16	0.89 (0.84–0.95)	<0.01	1.12 (0.77–1.62)	0.56
Region (vs. Northeast)								
Midwest	1.19 (1.13–1.26)	<0.01	1.07 (0.95–1.19)	0.28	1.22 (1.15–1.30)	<0.01	1.12 (0.91–1.39)	0.27
South	1.45 (1.38–1.52)	<0.01	1.16 (1.05–1.29)	<0.01	1.51 (1.43–1.59)	<0.01	1.60 (1.33–1.91)	<0.01
West and Other US territories	1.33 (1.26–1.40)	<0.01	1.15 (1.02–1.30)	0.03	1.38 (1.30–1.47)	<0.01	1.21 (0.95–1.55)	0.12
Primary disease leading to ESRD (vs. diabetes of any type)								
Hypertensive nephrosclerosis	0.99 (0.94–1.05)	0.73	0.96 (0.84–1.10)	0.56	0.99 (0.93–1.05)	0.67	1.10 (0.85–1.43)	0.45
Polycystic kidney disease	1.03 (0.95–1.12)	0.42	0.99 (0.83–1.19)	0.94	1.04 (0.95–1.15)	0.37	1.03 (0.72–1.47)	0.86
Focal glomerular sclerosis	0.99 (0.91–1.07)	0.75	0.90 (0.75–1.09)	0.29	1.04 (0.94–1.14)	0.44	0.76 (0.53–1.09)	0.13
Other	1.00 (0.94–1.06)	0.95	0.98 (0.86–1.12)	0.77	1.00 (0.94–1.07)	0.92	0.98 (0.76–1.26)	0.87
CCI ≥5 (vs. <5)	0.99 (0.94–1.04)	0.58	0.99 (0.88–1.11)	0.85	0.99 (0.94–1.05)	0.76	0.92 (0.75–1.14)	0.44
Comorbid health conditions (vs. absence of condition)								
Cardiovascular disease	1.01 (0.97–1.05)	0.73	0.95 (0.87–1.05)	0.33	1.02 (0.97–1.06)	0.42	1.02 (0.86–1.20)	0.82
Chronic pulmonary disease	1.01 (0.97–1.06)	0.66	1.09 (0.98–1.21)	0.1	0.99 (0.94–1.04)	0.68	1.03 (0.85–1.25)	0.74
Diabetes	0.98 (0.93–1.03)	0.39	1.05 (0.92–1.19)	0.47	0.96 (0.90–1.02)	0.18	1.04 (0.82–1.32)	0.75
Liver disease	0.92 (0.88–0.96)	<0.01	1.00 (0.90–1.12)	0.93	0.90 (0.86–0.95)	<0.01	0.93 (0.76–1.14)	0.49
Rheumatologic disease	1.03 (0.97–1.11)	0.33	1.11 (0.94–1.32)	0.20	1.03 (0.96–1.11)	0.43	0.88 (0.64–1.20)	0.41
Donor type deceased (vs. living)	1.15 (1.09–1.20)	<0.01	1.14 (1.02–1.28)	0.02	1.14 (1.07–1.20)	<0.01	1.20 (0.99–1.45)	0.06
Cold ischemia time <24 h (vs. ≥24 h)	0.97 (0.93–1.01)	0.19	0.93 (0.84–1.03)	0.17	0.97 (0.93–1.02)	0.29	1.01 (0.82–1.24)	0.92
Donor creatinine >1.5 mg/dL (vs. ≤1.5 mg/dL)	0.99 (0.95–1.03)	0.61	0.94 (0.84–1.04)	0.23	1.01 (0.96–1.06)	0.76	0.85 (0.69–1.04)	0.12
Time on dialysis prior to KT in years	0.99 (0.99–1.00)	0.06	1.01 (1.00–1.03)	0.05	0.99 (0.98–1.00)	<0.01	0.99 (0.97–1.02)	0.67
Wait time in years	0.99 (0.98–1.00)	<0.01	0.98 (0.96–1.00)	0.06	0.99 (0.98–1.00)	0.02	1.00 (0.96–1.04)	0.88
PRAs ≥80% (vs. <80%)	0.96 (0.91–1.02)	0.16	1.12 (0.95–1.32)	0.18	0.95 (0.89–1.01)	0.09	0.89 (0.67–1.18)	0.42
HLA A B donor-recipient match ≥3 (vs. <3)	0.97 (0.93–1.01)	0.1	1.00 (0.92–1.10)	0.92	0.95 (0.90–0.99)	0.02	1.10 (0.93–1.30)	0.26
Calendar year of transplant 2011–2013 (vs. 2014–2016)	0.51 (0.46–0.57)							
Induction immunosuppressive therapy^b^ (vs. absence of therapy)								
ATG	0.95 (0.91–1.00)	0.04	0.86 (0.77–0.95)	<0.01	0.96 (0.91–1.01)	0.13	1.15 (0.94–1.40)	0.18
Alemtuzumab	0.95 (0.89–1.00)	0.07	0.92 (0.81–1.06)	0.25	0.96 (0.90–1.03)	0.23	0.88 (0.70–1.12)	0.29
Basiliximab	0.97 (0.92–1.03)	0.31	0.99 (0.88–1.11)	0.82	0.95 (0.90–1.01)	0.12	1.11 (0.89–1.39)	0.36
Other immunosuppression	0.88 (0.74–1.04)	0.13	0.81 (0.48–1.35)	0.41	0.86 (0.72–1.04)	0.12	1.15 (0.63–2.09)	0.66
Maintenance immunosuppressive therapy^c^ (vs. absence of therapy)								
MMF	0.90 (0.82–0.98)	0.01	0.86 (0.72–1.04)	0.13	0.88 (0.79–0.97)	0.01	1.11 (0.78–1.56)	0.56
Tacrolimus	0.80 (0.73–0.87)	<0.01	0.85 (0.71–1.01)	0.07	0.77 (0.70–0.85)	<0.01	0.99 (0.68–1.44)	0.94
AZA, everolimus, and/or cyclosporine	0.89 (0.79–1.01)	0.07	0.86 (0.67–1.10)	0.22	0.88 (0.75–1.02)	0.09	1.37 (0.83–2.26)	0.22
Other immunosuppression	0.98 (0.90–1.06)	0.58	1.04 (0.87–1.24)	0.70	1.00 (0.91–1.10)	0.95	0.79 (0.54–1.16)	0.23
Prednisone or methylprednisolone	0.97 (0.90–1.05)	0.49	1.02 (0.86–1.22)	0.80	1.00 (0.92–1.10)	0.94	0.60 (0.45–0.81)	<0.01

Abbreviations: ATG, antithymocyte globulin; AZA, azathioprine; CCI, Charlson comorbidity index; CI, confidence interval; CMV, cytomegalovirus; D, donor; D+, seropositive donor; D–, seronegative donor; ESRD, end-stage renal disease; HLA, human leukocyte antigen; HR, hazard ratio; KT, kidney transplant; MMF, mycophenolate mofetil; PRA, panel-reactive antibody; R, recipient; R+, seropositive recipient; R–, seronegative recipient; US, United States.

^a^ Other includes Asian, American Indian, Alaska Native, Native Hawaiian, Pacific Islander, multiracial, other, and unknown.

^b^ Other immunosuppression therapies included daclizumab, muromonab-CD3, rituximab, and cyclophosphamide.

^c^ Other immunosuppression maintenance therapies included sirolimus, leflunomide, belatacept, or any other.

## Discussion

Based on a large cohort of adult KTRs who received their first KTs between July 2011 and December 2016, we found that most, but not all, high- and intermediate-risk KTRs used CMV prophylaxis. CMV prophylaxis was more common among high- (86.9%) than intermediate- (83.6%) and low-risk (31.7%) KTRs, with all those KTRs using valganciclovir and almost 60% of valganciclovir users using 450 mg per day. Furthermore, we found that the majority of KTRs used CMV prophylaxis for less than the guideline-recommended duration of 200 and 100 days for high- and intermediate-risk KTRs, respectively (4, 5, 7).

Compared with our current research, Santos *et al.* (2016) used USRDS-Medicare data for the period covering June 2006 to 2011 and found that 60% of KTRs used CMV prophylaxis (71%, 63%, and 34% of high-, intermediate- and low-risk KTRs, respectively) (13). In our study, we found, using more recent data from 2011 to 2017, that proportionately more KTRs—overall, high- and intermediate-risk KTRs—used prophylaxis, while low-risk KTRs continued to have the same proportion on prophylaxis. Overall, these findings reflect an improvement in adherence to guideline recommendations on the use of prophylaxis in high- and intermediate-risk KTRs, and a persistent overuse of prophylaxis in low-risk KTRs. Furthermore, we found that the mean duration of CMV prophylaxis was also longer in our study; however, still only approximately one in five high-risk KTRs completed 200 days of CMV prophylaxis and just over one in three intermediate-risk KTRs completed 100 days of CMV prophylaxis. These findings highlight premature discontinuation of CMV prophylaxis among high-and intermediate-risk KTRs.

To capture use of alternate treatments potentially still being used, the initial study definitions of CMV prophylaxis included treatment with valganciclovir, acyclovir, ganciclovir, valacyclovir, foscarnet, or cidofovir. Since current CMV treatment guidelines do not include the use of agents other than valganciclovir and ganciclovir, we utilized dose-based algorithms to identify alternative agents as CMV prophylaxis considering previous clinical guidelines and clinical expert inputs. Based on finding less than 0.5% of patients who received an alternative agent for CMV prophylaxis, we did not report findings due to lack of meaningful comparisons.

We also explored the impact of factors associated with the use and duration of CMV prophylaxis. In general, we found that characteristics thought or known to be risk factors for graft rejection and CMV infection/disease were key determinants for use, and longer duration, of CMV prophylaxis. The literature suggests that CMV serostatus (risk) and young age are risk factors for CMV (3, 6, 7). In addition, young age, high PRA, deceased donor, cold ischemia time >24 h, and HLA mismatch are also known risk factors for acute rejection requiring intensive immunosuppressive therapy (14). The use of certain T-cell depleting agents (ATG, alemtuzumab) (13, 15, 16) and high doses of immunosuppressive agents have been shown to be associated with increases in the risk of CMV (7). Additionally, younger age (17, 18), African American race, use of mammalian target of rapamycin inhibitors (19-24), and PRA ≥80% (25) are associated with decreased risk of CMV infection, and hence, decreased need for CMV prophylaxis. There is some evidence that basiliximab is negatively associated with CMV infection and the need for prophylaxis (17, 26). Our findings were mostly consistent in this regard. High-risk and younger (18–65 years) KTRs were the most likely to receive CMV prophylaxis and were the least likely to have discontinued CMV prophylaxis. Also consistent with previously published data, we found that KTRs who used basiliximab, AZA, everolimus, or cyclosporine, or other maintenance immunosuppressive agents, which included sirolimus (by high-risk KTRs), and whose kidney grafts spent <24 h in cold ischemia were less likely to have started prophylaxis. We also found that occurrence of myelosuppressive events was one of the factors, regardless of risk group, most strongly associated risk of CMV prophylaxis discontinuation. This finding is consistent with the prior studies highlighting valganciclovir discontinuation as a result of leukopenia and/or neutropenia. For example, Brar et al. (2021) recently reported that, among high-risk KTRs who received their KTs at a single institution, those who developed neutropenia were much more likely to have discontinued or reduced the dose of their prophylaxis as well as maintenance immunosuppressive therapies (27).

Retrospective database studies that use registry and claims data, such as ours, are inherently limited by the how recent the data are and by the specific types of information that are available, which are often obtained for purposes other than the study being designed. For our study, the data collected for surveillance and administrative purposes lacked clinical measures such as creatine levels or glomerular filtration rates (GFRs) collected at key points, such as initiation of CMV antiviral agents. The lack of clinical measures at precise times during treatment translated to limitations in understanding if the intended dose of valganciclovir for CMV prophylaxis was appropriate. Our methods used to impute TDD may not accurately reflect the intended dose of valganciclovir, as dose adjustments due to renal insufficiency or impairment were not available. We tested an alternative method to confirm intended dose by assuming centers would apply a uniform protocol for the use of CMV prophylaxis by CMV serostatus. However, the center level analysis showed variation in the use of valganciclovir dose and did not inform intended dose. Therefore, it is possible that those with renal impairment (i.e., low GFR) may have used valganciclovir 450 mg, rather than the intended dose of 900 mg. These limitations within the data may have led to misclassification errors; however, since the majority of KTRs received a well-functioning kidney, this situation may apply to only a small fraction of KTRs.

To ensure use of valganciclovir or ganciclovir use was correctly assigned as CMV prophylaxis instead of pre-emptive therapy in our analysis, in addition to identifying individuals who initiated valganciclovir or ganciclovir within 28 post KT, we excluded individuals with a diagnosis of CMV infection during the baseline study period. Given the mean length of stay for the kidney transplantation procedure ranges from 4.5 to 5.5 days and the mean (SD) time to initiate either ganciclovir or valganciclovir was 4.3 (4.5) days in our study, these agents seem to be initiated at discharge without a prior diagnosis of CMV during the index transplant (28). Therefore, it was highly unlikely that these agents were used as pre-emptive therapy. Finally, because we only included Medicare Part D enrollees in our sample, our findings may not be generalizable to commercial insured or Medicare advantage enrollees and individuals who reside outside the United States.

However, despite these limitations, our study has many strengths. Our study used a large and detailed database containing KT registry data linked to Medicare claims that allowed us to analyze a broad number of donor and recipient clinical characteristics. Furthermore, we were able to accurately capture medication use patterns by limiting the sample to Medicare Part D-covered beneficiaries. Our findings contribute to the literature by documenting improvements in adherence to guideline recommendations for managing CMV in KTRs.

## Conclusion

This study provides the most up-to-date information on national-level CMV prophylaxis among KTRs in the US. Most, but not all, high- and intermediate-risk KTRs received CMV prophylaxis, and virtually all KTRs who started prophylaxis used valganciclovir. However, our findings also highlight that adherence to the recommended duration of CMV prophylaxis is suboptimal. Furthermore, this is the first study of a very large sample of KTRs to confirm the association between development of leukopenia and neutropenia and subsequent risk of CMV prophylaxis discontinuation.

## Data Availability

This study used data from the USRDS-Medicare database, which was provided to the study team subject to the terms of data use agreement (DUA) 2020-41f. The data are not publicly available due to privacy laws and cannot be shared by the authors. However, data obtained from the USRDS-Medicare database for this study may be accessed by applying to USRDS/NIDDK/CMS at usrds@niddk.nih.gov. Upon request, the corresponding author will provide the original data request and the programs used to derive this study’s analytic cohort.
