# Expression of Concern: Real-World Treatment Patterns of Antiviral Prophylaxis for Cytomegalovirus Among Adult Kidney Transplant Recipients: A Linked USRDS-Medicare Database Study

**DOI:** 10.3389/ti.2023.12367

**Published:** 2023-11-10

**Authors:** Transplant International Editorial Office

With this notice, Transplant International states its awareness of concerns as raised by the authors regarding the data validity of “Real-World Treatment Patterns of Antiviral Prophylaxis for Cytomegalovirus Among Adult Kidney Transplant Recipients: A Linked USRDS-Medicare Database Study” published on 12 August 2022. While a revised version is currently being conducted by the authors in collaboration with the Editorial Office, this expression of concern has been posted while Transplant International awaits the revisions. It will be updated accordingly after that time.

UPDATE: This article has now been corrected. Please find the full corrigendum here: 10.3389/ti.2024.11921.

