# Corrigendum: Real-World Treatment Patterns of Antiviral Prophylaxis for Cytomegalovirus Among Adult Kidney Transplant Recipients: A Linked USRDS-Medicare Database Study

**DOI:** 10.3389/ti.2024.11921

**Published:** 2024-02-14

**Authors:** Amit D. Raval, Michael L. Ganz, Kathy Fraeman, Andrea L. Lorden, Shanmugapriya Saravanan, Yuexin Tang, Carlos A. Q. Santos

**Affiliations:** ^1^ Merck & Co., Inc., Kenilworth, NJ, United States; ^2^ Evidera, Bethesda, MD, United States; ^3^ Rush Medical College, Rush University, Chicago, IL, United States

**Keywords:** kidney transplantation, antiviral, cytomegalovirus, prophylaxis, pharmacoepidemiology

## Abstract

**Figure:**
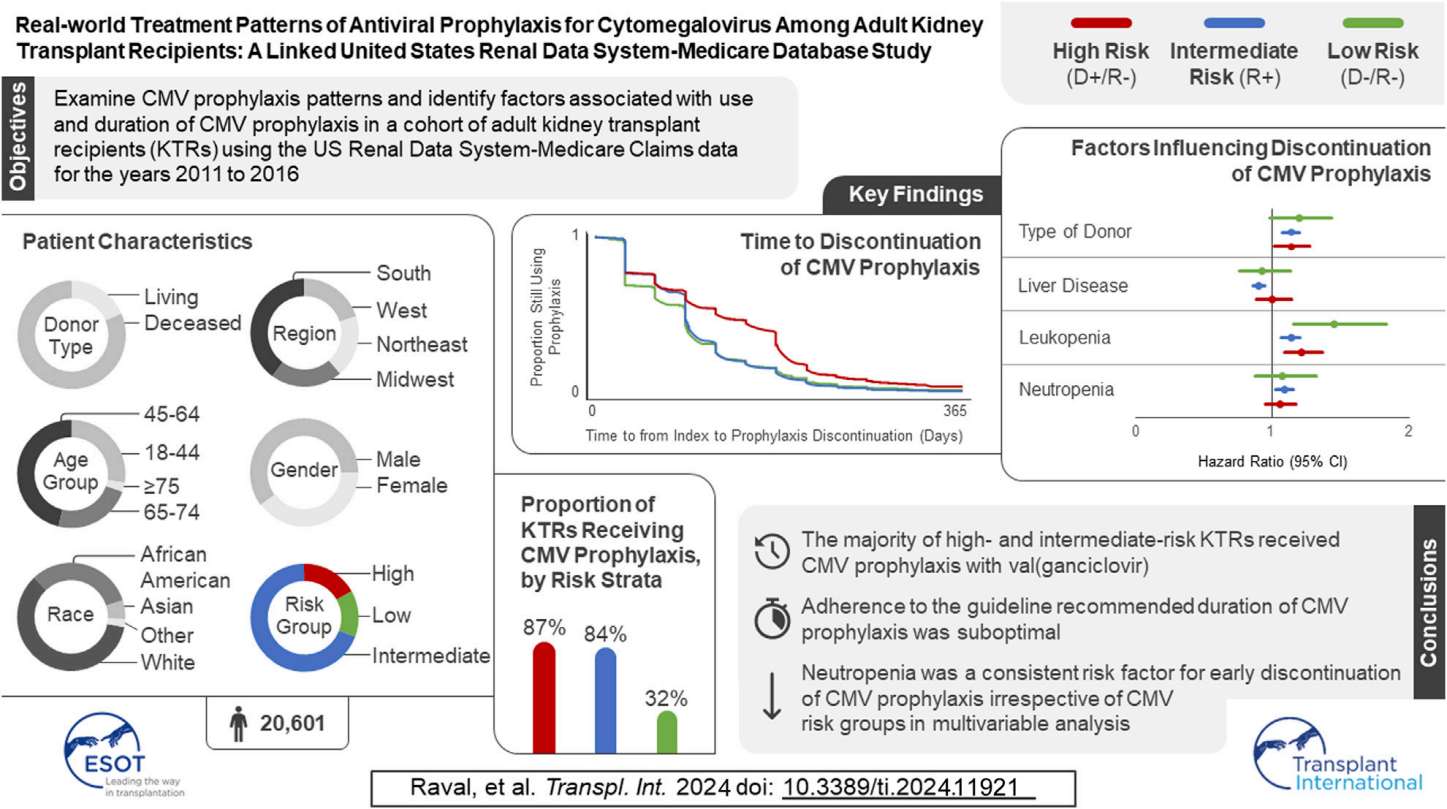

In the original article, there was a mistake in the Graphical Abstract as published. The number of participants in the study sample has changed, along with minor changes to the proportion of KTRs receiving CMV prophylaxis by risk strata, time to discontinuation of CMV prophylaxis, and the factors influencing discontinuation of CMV prophylaxis. The corrected Graphical Abstract appears below.

In the original article, there was a mistake in Figure 1 as published. Programing errors in the cohort selection led to differences in the cohort selected along with minor changes to the patient attrition related to inclusion and exclusion criteria. The corrected Figure 1 appears below.

**FIGURE 1:**
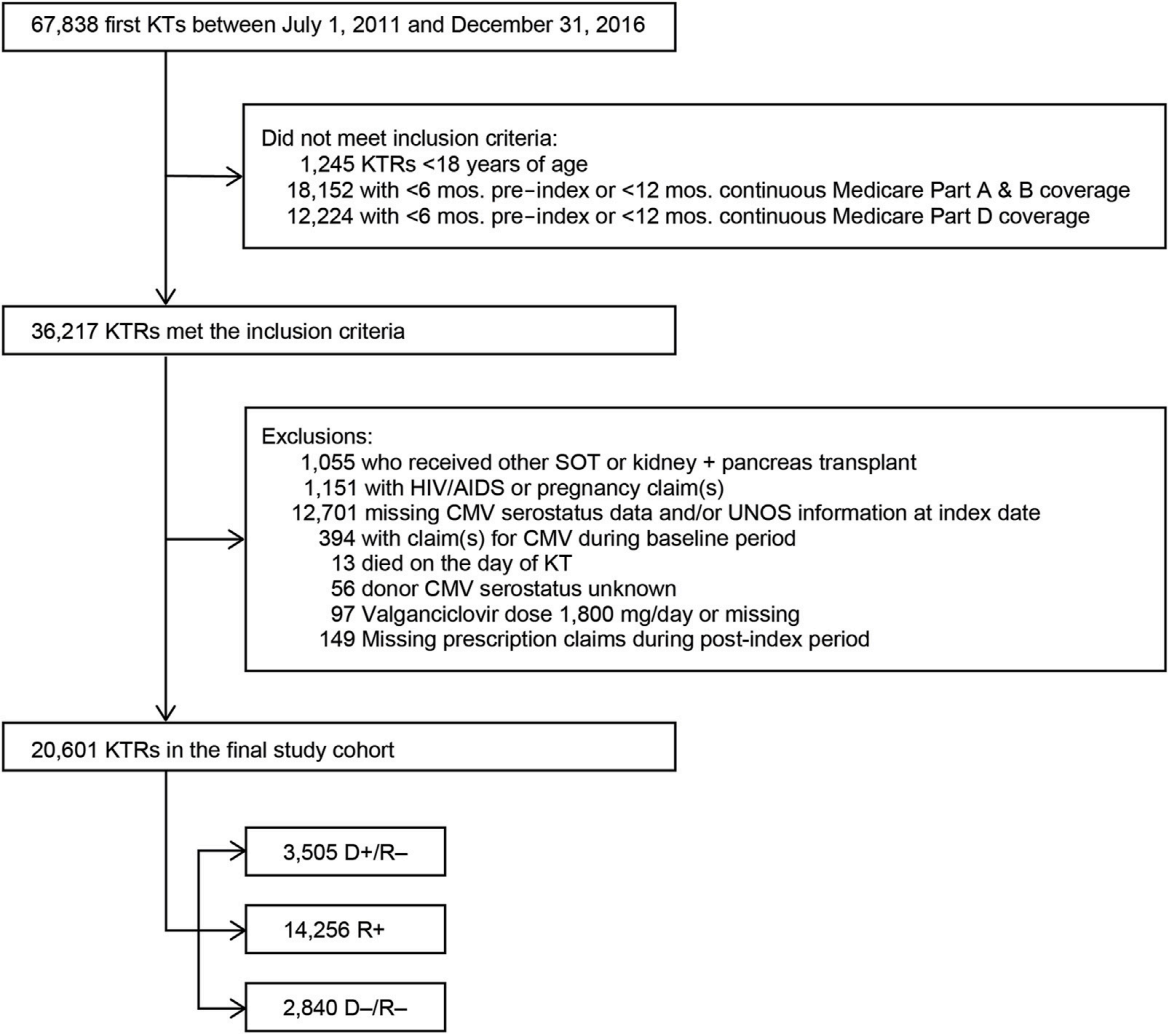
Study sample selection. Abbreviations: AIDS, acquired immunodeficiency syndrome; CMV, cytomegalovirus; D+, seropositive donor; D–, seronegative donor; HIV, human immunodeficiency virus; KT, kidney transplant; KTRs, kidney transplant recipients; mos., months; R+, seropositive recipient; R–, seronegative recipient; SOT, solid organ transplant.

In the original article, there was a mistake in Figure 2 as published. Changes in the composition of the study cohort due to programming changes resulted in slightly different KM curves. The corrected Figure 2 appears below.

**FIGURE 2:**
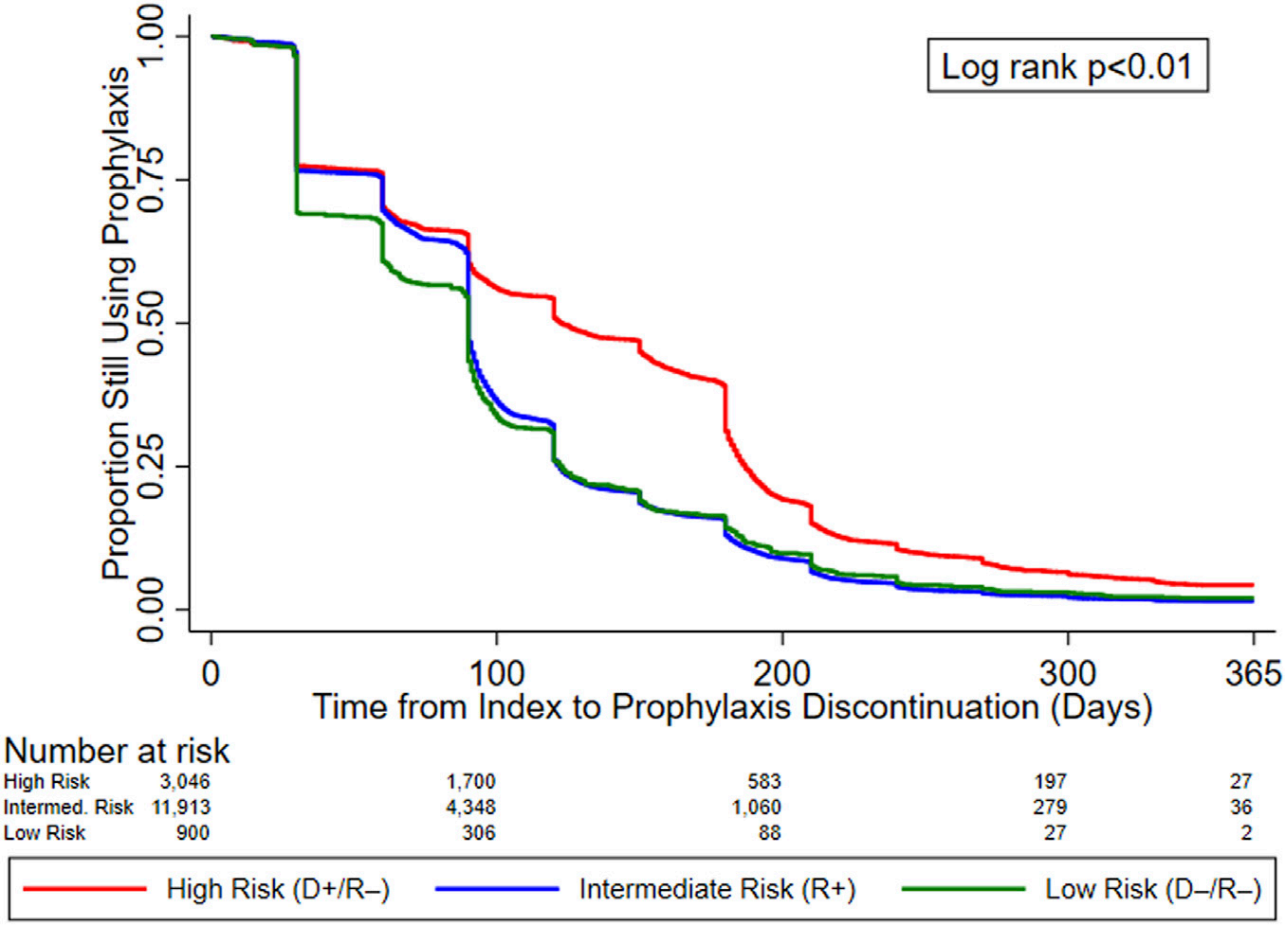
KM curves for time to prophylaxis discontinuation, by serostatus (CMV Risk Group). Abbreviations: D+, seropositive donor; D–, seronegative donor; R+, seropositive recipient; R–, seronegative recipient.

In the original article, there was a mistake in Table 1 as published. Changes in the composition of the study cohort due to programming errors that were corrected resulted in different numbers of patients reported throughout the table and slight differences in the proportions of patients in the various subgroups reported. The corrected Table 1 appears below.

**TABLE 1:** Baseline demographic, clinical, and medication-related characteristics of adult KTRs.

Characteristic	Overall (*N* = 20,601)	High risk (D+/R–) (*N* = 3,505)	Intermediate risk (R+) (*N* = 14,256)	Low risk (D–/R–) (*N* = 2,840)	*p*-value^a^
Mean age in years (SD)	53.2 (14.0)	51.7 (14.6)	53.9 (13.6)	51.4 (15.0)	<0.01
Age category in years, *N* (%)					
18–44	5,670 (27.5%)	1,102 (31.4%)	3,601 (25.3%)	967 (34.0%)	<0.01
45–64	9,545 (46.3%)	1,538 (43.9%)	6,862 (48.1%)	1,145 (40.3%)
65–74	4,837 (23.5%)	779 (22.2%)	3,400 (23.8%)	658 (23.2%)	
≥75	549 (2.7%)	86 (2.5%)	393 (2.8%)	70 (2.5%)	
Gender, *N* (%)					
Male	12,383 (60.1%)	2,467 (70.4%)	7,951 (55.8%)	1,965 (69.2%)	<0.01
Female	8,218 (39.9%)	1,038 (29.6%)	6,305 (44.2%)	875 (30.8%)	
Race, *N* (%)					
White	12,366 (60.0%)	2,479 (70.7%)	7,757 (54.4%)	2,130 (75.0%)	<0.01
African American	6,600 (32.0%)	932 (26.6%)	5,029 (35.3%)	639 (22.5%)	
Asian	1,147 (5.6%)	50 (1.4%)	1,060 (7.4%)	37 (1.3%)	
Other^b^					
Hispanic ethnicity, *N* (%)					
Yes	4,346 (21.1%)	435 (12.4%)	3,642 (25.5%)	269 (9.5%)	<0.01
No	16,093 (78.1%)	3,037 (86.6%)	10,504 (73.7%)	2,552 (89.9%)	
Unknown	162 (0.8%)	33 (0.9%)	110 (0.8%)	19 (0.7%)	
Geographic region, *N* (%)					
Northeast	3,830 (18.6%)	720 (20.5%)	2,406 (16.9%)	704 (24.8%)	<0.01
Midwest	4,424 (21.5%)	815 (23.3%)	2,822 (19.8%)	787 (27.7%)	
South	8,156 (39.6%)	1,377 (39.3%)	5,869 (41.2%)	910 (32.0%)	
West	4,137 (20.1%)	589 (16.8%)	3,123 (21.9%)	425 (15.0%)	
Other US territories	54 (0.3%)	<11	36 (0.3%)	14 (0.5%)	
Primary diagnosis leading to ESRD, *N* (%)					
Diabetes mellitus, Type 2	5,843 (28.4%)	873 (24.9%)	4,343 (30.5%)	627 (22.1%)	<0.01
Hypertensive nephrosclerosis	5,724 (27.8%)	863 (24.6%)	4,130 (29.0%)	731 (25.7%)	
Polycystic kidney disease	1,289 (6.3%)	255 (7.3%)	826 (5.8%)	208 (7.3%)	
Focal glomerular sclerosis	1,157 (5.6%)	221 (6.3%)	761 (5.3%)	175 (6.2%)
Systemic lupus erythematosus	751 (3.6%)	108 (3.1%)	561 (3.9%)	82 (2.9%)	
Diabetes mellitus - Type I	720 (3.5%)	146 (4.2%)	427 (3.0%)	147 (5.2%)	
IGA nephropathy	669 (3.2%)	125 (3.6%)	421 (3.0%)	123 (4.3%)	
Chronic glomerulonephritis unspecified	502 (2.4%)	88 (2.5%)	343 (2.4%)	71 (2.5%)	
Malignant hypertension	250 (1.2%)	46 (1.3%)	174 (1.2%)	30 (1.1%)	
Membranous glomerulonephritis	199 (1.0%)	47 (1.3%)	126 (0.9%)	26 (0.9%)	
Other Disease	3,497 (17.0%)	733 (20.9%)	2,144 (15.0%)	620 (21.8%)	
Charlson Comorbidity Index, *N* (%)					
0	0 (0.0%)	0 (0.0%)	0 (0.0%)	0 (0.0%)	<0.01
1–2	4,683 (22.7%)	839 (23.9%)	3,114 (21.8%)	730 (25.7%)	
3–4	8,110 (39.4%)	1,438 (41.0%)	5,525 (38.8%)	1,147 (40.4%)	
≥5	7,808 (37.9%)	1,228 (35.0%)	5,617 (39.4%)	963 (33.9%)	
Comorbid health conditions, *N* (%)					
Congestive heart failure	4,912 (23.8%)	782 (22.3%)	3,483 (24.4%)	647 (22.8%)	0.01
Diabetes	9,091 (44.1%)	1,441 (41.1%)	6,565 (46.1%)	1,085 (38.2%)	<0.01
Diabetes without chronic complication	3,948 (19.2%)	635 (18.1%)	2,802 (19.7%)	511 (18.0%)	0.03
Diabetes with chronic complication	8,586 (41.7%)	1,358 (38.7%)	6,220 (43.6%)	1,008 (35.5%)	<0.01
Chronic pulmonary disease	3,345 (16.2%)	587 (16.7%)	2,288 (16.0%)	470 (16.5%)	0.54
Peripheral vascular disease	5,025 (24.4%)	849 (24.2%)	3,575 (25.1%)	601 (21.2%)	<0.01
Rheumatologic disease	1,389 (6.7%)	208 (5.9%)	1,016 (7.1%)	165 (5.8%)	<0.01
Mild to moderate liver disease	3,016 (14.6%)	482 (13.8%)	2,147 (15.1%)	387 (13.6%)	0.04
Sever liver disease	91 (0.4%)	<11	75 (0.5%)	<11	0.02
Myocardial infarction	1,843 (8.9%)	307 (8.8%)	1,275 (8.9%)	261 (9.2%)	0.84
Dementia	164 (0.8%)	38 (1.1%)	96 (0.7%)	30 (1.1%)	0.01
Mean time on dialysis prior to KT (SD), years	4.8 (3.2)	4.6 (3.1)	4.9 (3.3)	4.1 (3.1)	<0.01
Mean wait time (SD), years	2.6 (2.1)	2.5 (2.1)	2.6 (2.2)	2.2 (1.9)	<0.01
PRA, *N* (%)					
0%	13,565 (65.8%)	2,498 (71.3%)	9,066 (63.6%)	2,001 (70.5%)	<0.01
1%–19%	1,791 (8.7%)	308 (8.8%)	1,240 (8.7%)	243 (8.6%)	
20%–79%	3,100 (15.0%)	464 (13.2%)	2,255 (15.8%)	381 (13.4%)	
80%–100%	1,898 (9.2%)	196 (5.6%)	1,555 (10.9%)	147 (5.2%)	
Missing	247 (1.2%)	39 (1.1%)	140 (1.0%)	68 (2.4%)	
HLA A B donor-recipient match, *N* (%)					
0	4,338 (21.1%)	701 (20.0%)	3,086 (21.6%)	551 (19.4%)	<0.01
1	6,872 (33.4%)	1,217 (34.7%)	4,778 (33.5%)	877 (30.9%)	
2	4,553 (22.1%)	777 (22.2%)	3,127 (21.9%)	649 (22.9%)	
≥3	4,601 (22.3%)	766 (21.9%)	3,104 (21.8%)	731 (25.7%)	
Missing	237 (1.2%)	44 (1.3%)	161 (1.1%)	32 (1.1%)	
Hepatitis C seropositive, *N* (%)	849 (4.1%)	108 (3.1%)	648 (4.5%)	93 (3.3%)	<0.01
Epstein-Barr virus antibody positive, *N* (%)	16,887 (82.0%)	2,737 (78.1%)	11,864 (83.2%)	2,286 (80.5%)	<0.01
Calendar year of transplant, *N* (%)					
2011	1,857 (9.0%)	338 (9.6%)	1,287 (9.0%)	232 (8.2%)	<0.01
2012	3,613 (17.5%)	607 (17.3%)	2,523 (17.7%)	483 (17.0%)	
2013	3,552 (17.2%)	598 (17.1%)	2,515 (17.6%)	439 (15.5%)	
2014	3,516 (17.1%)	609 (17.4%)	2,441 (17.1%)	466 (16.4%)	
2015	3,950 (19.2%)	659 (18.8%)	2,679 (18.8%)	612 (21.5%)	
2016	4,113 (20.0%)	694 (19.8%)	2,811 (19.7%)	608 (21.4%)	
Used immunosuppressive agents, *N* (%)	20,376 (98.9%)	3,466 (98.9%)	14,092 (98.8%)	2,818 (99.2%)	0.21
Induction immunosuppressive therapy, *N* (%)					
ATG	11,148 (54.7%)	1,801 (52.0%)	7,808 (55.4%)	1,539 (54.6%)	<0.01
Basiliximab	4,518 (22.2%)	805 (23.2%)	3,114 (22.1%)	599 (21.3%)	0.16
Alemtuzumab	3,369 (16.5%)	600 (17.3%)	2,316 (16.4%)	453 (16.1%)	0.36
Rituximab	142 (0.7%)	12 (0.3%)	117 (0.8%)	13 (0.5%)	<0.01
Muromonab-CD3	20 (0.10%)	<11	<11	<11	0.02
Daclizumab	<11	0 (0.0%)	<11	0 (0.0%)	NA
Cyclophosphamide					
Maintenance immunosuppressive therapy, *N* (%)					
Prednisone or methylprednisolone	19,623 (96.3%)	3,320 (95.8%)	13,595 (96.5%)	2,708 (96.1%)	0.13
MMF	19,624 (96.3%)	3,328 (96.0%)	13,613 (96.6%)	2,683 (95.2%)	<0.01
Tacrolimus	19,327 (94.9%)	3,272 (94.4%)	13,383 (95.0%)	2,672 (94.8%)	0.40
Belatacept	530 (2.6%)	89 (2.6%)	381 (2.7%)	60 (2.1%)	0.21
Cyclosporine	399 (2.0%)	70 (2.0%)	275 (2.0%)	54 (1.9%)	0.95
Sirolimus	239 (1.2%)	45 (1.3%)	144 (1.0%)	50 (1.8%)	<0.01
Everolimus	207 (1.0%)	44 (1.3%)	125 (0.9%)	38 (1.3%)	0.02
Leflunomide	11 (0.05%)	<11	<11	<11	0.72
AZA	65 (0.3%)	12 (0.3%)	42 (0.3%)	11 (0.4%)	0.70
Other	338 (1.7%)	53 (1.5%)	248 (1.8%)	37 (1.3%)	0.19
Donor type, *N* (%)					
Deceased	16,789 (81.5%)	2,907 (82.9%)	11,866 (83.2%)	2,016 (71.0%)	<0.01
Living	3,812 (18.5%)	598 (17.1%)	2,390 (16.8%)	824 (29.0%)	
Mean cold ischemia time in hours (SD)	14.9 (10.0)	14.7 (9.6)	15.4 (10.0)	12.9 (9.9)	<0.01
Cold ischemia time in hours category, *N* (%)					
<24 h	16,807 (81.6%)	2,896 (82.6%)	11,514 (80.8%)	2,397 (84.4%)	<0.01
≥24 h	3,443 (16.7%)	551 (15.7%)	2,537 (17.8%)	355 (12.5%)	
Missing	351 (1.7%)	58 (1.7%)	205 (1.4%)	88 (3.1%)	
Mean donor creatinine in mg/dL (SD)	1.1 (1.0)	1.1 (1.1)	1.1 (0.9)	1.1 (0.9)	0.03
Donor creatinine in mg/dL category, *N* (%)					
≤1.5 mg/dL	17,187 (83.4%)	2,935 (83.7%)	11,817 (82.9%)	2,435 (85.7%)	<0.01
>1.5 mg/dL	3,399 (16.5%)	568 (16.2%)	2,429 (17.0%)	402 (14.2%)	
Missing	15 (0.07%)	<11	<11	<11	

Abbreviations: ATG, antithymocyte globulin; AZA, azathioprine; D, donor; D+, seropositive donor; D–, seronegative donor; ESRD, end-stage renal disease; HLA, human leukocyte antigen; IGA, immunoglobulin A; KT, kidney transplant; MMF, mycophenolate mofetil; NA, not applicable; PRA, panel-reactive antibody; R, recipient; R+, seropositive recipient; R–, seronegative recipient; SD, standard deviation; US, United States.

^a^ *p*-values are compared across patients by donor/recipient serostatus group using t-tests or analysis of variance (ANOVA) for continuous variables or chi-square tests for categorical variables.

^b^ Other includes American Indian, Alaska Native, Native Hawaiian, Pacific Islander, multiracial, other, and unknown.

In the original article, there was a mistake in Table 2 as published. Changes in the composition of the study cohort due to programming errors that were corrected resulted in different numbers of patients reported throughout the table and slight differences in the proportions of patients in the various subgroups reported. The corrected Table 2 appears below.

**TABLE 2:** Characteristics of CMV prophylaxis among adults undergoing first kidney transplant by serostatus.

Prophylaxis Information	Overall (*N* = 20,601)	High risk (D+/R–) (*N* = 3,505)	Intermediate risk (R+) (*N* = 14,256)	Low risk (D–/R–) (*N* = 2,840)	*p*-value
All prophylaxis agents					
CMV prophylaxis					
No prophylaxis	4,742 (23.0%)	459 (13.1%)	2,343 (16.4%)	1,940 (68.3%)	<0.01
Prophylaxis	15,859 (77.0%)	3,046 (86.9%)	11,913 (83.6%)	900 (31.7%)	
Type of prophylaxis, *N* (%)					
Valganciclovir	15,859 (100.0%)	3,046 (100.0%)	11,913 (100.0%)	900 (100.0%)	NA
Index dose 450 mg	9,462 (59.7%)	1,450 (47.6%)	7,518 (63.1%)	494 (54.9%)	<0.01
Index dose 900 mg	5,153 (32.5%)	1,371 (45.0%)	3,461 (29.1%)	321 (35.7%)	
Other index dose	1,244 (7.8%)	225 (7.4%)	934 (7.8%)	85 (9.4%)	
Ganciclovir					
Mean time to initiate any CMV prophylaxis in days (SD)	4.2 (4.4)	4.5 (4.7)	4.1 (4.3)	4.4 (4.9)	<0.01
Mean duration of CMV prophylaxis in days (SD)	107.5 (74.4)	134.1 (90.5)	101.5 (68.1)	97.6 (74.1)	<0.01
Duration of CMV prophylaxis, *N* (%)					
≥72 days	10,297 (64.9%)	2,034 (66.8%)	7,752 (65.1%)	511 (56.8%)	<0.01
≥90 days	9,912 (62.5%)	1,986 (65.2%)	7,433 (62.4%)	493 (54.8%)	<0.01
≥100 days	6,359 (40.1%)	1,700 (55.8%)	4,352 (36.5%)	307 (34.1%)	<0.01
≥180 days	3,201 (20.2%)	1,187 (39.0%)	1,868 (15.7%)	146 (16.2%)	<0.01
≥200 days	1,733 (10.9%)	583 (19.1%)	1,062 (8.9%)	88 (9.8%)	<0.01
Valganciclovir 450 mg					
Mean time to initiate valganciclovir 450 mg prophylaxis in days (SD)	4.0 (4.2)	4.4 (4.8)	3.9 (4.1)	4.4 (4.8)	<0.01
Mean duration of valganciclovir 450 mg prophylaxis in days (SD)	115.2 (75.4)	151.0 (91.0)	108.7 (69.7)	109.6 (79.8)	<0.01
Duration of valganciclovir 450 mg prophylaxis, *N* (%)					
≥72 days	6,786 (71.7%)	1,093 (75.4%)	5,376 (71.5%)	317 (64.2%)	<0.01
≥90 days	6,587 (69.6%)	1,072 (73.9%)	5,206 (69.2%)	309 (62.6%)	<0.01
≥100 days	4,123 (43.6%)	928 (64.0%)	2,998 (39.9%)	197 (39.9%)	<0.01
≥180 days	2,151 (22.7%)	691 (47.7%)	1,357 (18.1%)	103 (20.9%)	<0.01
≥200 days	1,151 (12.2%)	340 (23.4%)	749 (10.0%)	62 (12.6%)	<0.01
Valganciclovir 900 mg					
Mean time to initiate valganciclovir 900 mg prophylaxis in days (SD)	3.9 (4.3)	4.2 (4.5)	3.8 (4.2)	3.7 (4.4)	0.02
Mean duration of valganciclovir 900 mg prophylaxis in days (SD)	87.7 (67.8)	111.8 (84.5)	79.6 (58.5)	72.3 (55.6)	<0.01
Duration of valganciclovir 900 mg prophylaxis, *N* (%)					
≥72 days	2,513 (48.8%)	760 (55.4%)	1,622 (46.9%)	131 (40.8%)	<0.01
≥90 days	2,413 (46.8%)	739 (53.9%)	1,544 (44.6%)	130 (40.5%)	<0.01
≥100 days	1,484 (28.8%)	614 (44.8%)	807 (23.3%)	63 (19.6%)	<0.01
≥180 days	716 (13.9%)	392 (28.6%)	305 (8.8%)	19 (5.9%)	<0.01
≥200 days	361 (7.0%)	174 (12.7%)	177 (5.1%)	<11	<0.01
Valganciclovir other dose					
Mean time to initiate valganciclovir other dose in days (SD)	6.6 (5.3)	6.9 (5.3)	6.5 (5.2)	6.8 (6.4)	0.57
Mean duration of valganciclovir other dose prophylaxis in days (SD)	131.5 (74.9)	160.9 (92.0)	125.1 (68.4)	123.7 (74.5)	<0.01
Duration of valganciclovir other dose prophylaxis, *N* (%)					
≥72 days	998 (80.2%)	181 (80.4%)	754 (80.7%)	63 (74.1%)	0.34
≥90 days	912 (73.3%)	175 (77.8%)	683 (73.1%)	54 (63.5%)	0.04
≥100 days	752 (60.5%)	158 (70.2%)	547 (58.6%)	47 (55.3%)	<0.01
≥180 days	334 (26.8%)	104 (46.2%)	206 (22.1%)	24 (28.2%)	<0.01
≥200 days	221 (17.8%)	69 (30.7%)	136 (14.6%)	16 (18.8%)	<0.01

Abbreviations: CMV, cytomegalovirus; D, donor; D+, seropositive donor; D–, seronegative donor; R, recipient; R+, seropositive recipient; R–, seronegative recipient; SD, standard deviation.

In the original article, there was a mistake in Table 3 as published. Changes in the composition of the study cohort due to programming errors that were corrected resulted in different coefficients and confidence intervals for the variables included in the regression. While a few relationships changed, those that did change did not influence or create a need to revise the conclusions of the study. The corrected Table 3 appears below.

**TABLE 3:** Logistic regression for probability of starting CMV prophylaxis among adults undergoing a first kidney transplant.

Predictors	Overall		High risk (D+/R–)		Intermediate risk (R+)		Low risk (D–/R–)	
	OR (95% CI)	*p*-value	OR (95% CI)	*p*-value	OR (95% CI)	*p*-value	OR (95% CI)	*p*-value
CMV serostatus (vs. D–/R–)								
D+/R–	17.16 (15.04–19.59)	<0.01						
R+	11.49 (10.42–12.67)	<0.01						
Age 18–64 years (vs. age ≥65)	1.61 (1.48–1.76)	<0.01	1.91 (1.52–2.40)	<0.01	1.64 (1.48–1.82)	<0.01	1.36 (1.10–1.69)	<0.01
Female gender (vs. male)	1.15 (1.06–1.25)	<0.01	1.01 (0.80–1.28)	0.90	1.19 (1.08–1.32)	<0.01	1.22 (1.00–1.47)	0.05
Race (vs. White)								
African American	1.15 (1.05–1.26)	<0.01	1.36 (1.04–1.78)	0.08	1.11 (0.99–1.24)	0.08	1.12 (0.90–1.39)	0.29
Other^a^	1.55 (1.32–1.82)	<0.01	2.25 (0.97–5.25)	0.06	1.42 (1.20–1.69)	<0.01	1.95 (1.18–3.22)	<0.01
Region (vs. Northeast)								
Midwest	0.55 (0.49–0.62)	<0.01	0.71 (0.53–0.96)	0.03	0.41 (0.35–0.48)	<0.01	0.66 (0.52–0.84)	<0.01
South	0.85 (0.76–0.95)	<0.01	0.85 (0.64–1.14)	0.28	0.60 (0.51–0.70)	<0.01	1.63 (1.30–2.03)	<0.01
West and Other US territories	0.85 (0.75–0.97)	0.02	1.10 (0.77–1.56)	0.61	0.69 (0.58–0.82)	<0.01	0.85 (0.65–1.13)	0.27
Primary disease leading to ESRD (vs. diabetes of any type)								
Hypertensive nephrosclerosis	1.18 (1.03–1.34)	0.02	0.99 (0.70–1.39)	0.94	1.32 (1.12–1.56)	<0.01	0.98 (0.72–1.34)	0.92
Polycystic kidney disease	1.06 (0.88–1.28)	0.54	0.95 (0.59–1.52)	0.82	1.17 (0.92–1.49)	0.19	0.86 (0.57–1.31)	0.49
Focal glomerular sclerosis	1.15 (0.95–1.40)	0.16	1.34 (0.76–2.37)	0.30	1.26 (0.98–1.62)	0.07	0.82 (0.53–1.26)	0.37
Other	1.09 (0.95–1.25)	0.23	0.90 (0.64–1.27)	0.56	1.27 (1.07–1.51)	<0.01	0.81 (0.59–1.10)	0.18
CCI ≥5 (vs. <5)	1.10 (0.98–1.23)	0.12	1.04 (0.76–1.41)	0.82	1.13 (0.98–1.31)	0.10	1.03 (0.79–1.33)	0.82
Comorbid health conditions								
Cardiovascular disease	0.94 (0.86–1.03)	0.18	0.89 (0.70–1.14)	0.35	0.90 (0.81–1.01)	0.07	1.11 (0.91–1.35)	0.32
Chronic pulmonary disease	0.91 (0.82–1.01)	0.07	1.02 (0.77–1.35)	0.89	0.83 (0.73–0.95)	<0.01	1.04 (0.83–1.31)	0.72
Diabetes	1.12 (0.99–1.27)	0.08	0.75 (0.54–1.04)	0.09	1.27 (1.08–1.50)	<0.01	1.05 (0.79–1.40)	0.73
Liver disease	1.05 (0.94–1.18)	0.37	1.09 (0.80–1.49)	0.59	1.04 (0.91–1.20)	0.54	1.02 (0.80–1.31)	0.85
Rheumatologic disease	0.88 (0.75–1.03)	0.10	0.92 (0.58–1.45)	0.71	0.87 (0.72–1.05)	0.15	0.88 (0.60–1.27)	0.48
Donor type deceased (vs. living)	0.91 (0.81–1.01)	0.07	0.82 (0.61–1.11)	0.20	0.93 (0.81–1.06)	0.28	0.93 (0.75–1.16)	0.54
Cold ischemia time <24 h (vs. ≥24 h)	0.95 (0.86–1.06)	0.37	1.18 (0.89–1.55)	0.26	0.92 (0.81–1.05)	0.23	0.89 (0.69–1.15)	0.38
Donor creatinine >1.5 mg/dL (vs. ≤1.5 mg/dL)	1.21 (1.09–1.36)	<0.01	1.22 (0.91–1.64)	0.19	1.21 (1.06–1.40)	<0.01	1.13 (0.88–1.45)	0.34
Time on dialysis prior to KT in years	1.02 (1.01–1.04)	<0.01	1.01 (0.97–1.06)	0.53	1.02 (1.01–1.04)	0.01	1.02 (0.99–1.06)	0.16
Wait time in years	0.99 (0.97–1.02)	0.62	1.00 (0.95–1.06)	0.98	0.99 (0.97–1.02)	0.47	1.01 (0.96–1.06)	0.77
PRAs ≥80% (vs. <80%)	1.36 (1.17–1.59)	<0.01	1.73 (1.00–2.98)	0.05	1.29 (1.08–1.55)	<0.01	1.33 (0.92–1.94)	0.13
HLA A B donor-recipient match ≥3 (vs. <3)	0.95 (0.87–1.04)	0.28	1.07 (0.83–1.37)	0.60	0.91 (0.81–1.02)	0.11	0.98 (0.80–1.20)	0.86
Calendar year of KT 2011–2013 (vs. 2014–2016)	1.12 (1.04–1.20)	<0.01	1.10 (0.90–1.35)	0.37	1.28 (1.17–1.41)	<0.01	0.73 (0.62–0.87)	<0.01
Induction immunosuppressive therapy^b^ (vs. absence of therapy)								
ATG	1.77 (1.59–1.97)	<0.01	1.18 (0.89–1.57)	0.26	2.04 (1.79–2.32)	<0.01	1.62 (1.26–2.07)	<0.01
Alemtuzumab	1.55 (1.36–1.78)	<0.01	1.06 (0.74–1.52)	0.73	1.67 (1.41–1.97)	<0.01	1.60 (1.19–2.16)	<0.01
Basiliximab	0.79 (0.70–0.88)	<0.01	0.97 (0.71–1.32)	0.84	0.71 (0.62–0.81)	<0.01	1.02 (0.78–1.35)	0.87
Other immunosuppression	1.69 (1.03–2.77)	0.04	0.69 (0.19–2.49)	0.57	1.39 (0.78–2.46)	0.27	4.48 (1.67–12.05)	<0.01
Maintenance immunosuppressive therapy^c^ (vs. absence of therapy)								
MMF	1.07 (0.88–1.30)	0.5	0.73 (0.42–1.25)	0.25	1.39 (1.10–1.76)	<0.01	0.81 (0.53–1.25)	0.34
Tacrolimus	1.16 (0.96–1.40)	0.11	0.96 (0.59–1.57)	0.87	1.50 (1.20–1.88)	<0.01	0.56 (0.36–0.87)	0.01
AZA, everolimus, and/or cyclosporine	0.37 (0.29–0.46)	<0.01	0.77 (0.41–1.47)	0.43	0.34 (0.26–0.44)	<0.01	0.56 (0.30–1.04)	0.07
Other immunosuppression	1.14 (0.95–1.37)	0.15	0.74 (0.46–1.17)	0.20	1.53 (1.22–1.92)	<0.01	0.71 (0.46–1.09)	0.12
Prednisone or methylprednisolone	0.54 (0.44–0.66)	<0.01	1.28 (0.82–1.98)	0.28	0.38 (0.28–0.51)	<0.01	0.52 (0.35–0.76)	<0.01

Abbreviations: ATG, antithymocyte globulin; AZA, azathioprine; CCI, Charlson comorbidity index; CI, confidence interval; CMV, cytomegalovirus; D, donor; D+, seropositive donor; D–, seronegative donor; ESRD, end-stage renal disease; HLA, human leukocyte antigen; KT, kidney transplant; MMF, mycophenolate mofetil; OR, odds ratio; PRA, panel-reactive antibody; R, recipient; R+, seropositive recipient; R–, seronegative recipient; US, United States.

^a^ Other includes Asian, American Indian, Alaska Native, Native Hawaiian, Pacific Islander, multiracial, other, and unknown.

^b^ Other immunosuppression therapies included daclizumab, muromonab-CD3, rituximab, and cyclophosphamide.

^c^ Other immunosuppression maintenance therapies included sirolimus, leflunomide, belatacept, or any other.

In the original article, there was a mistake in Table 4 as published. Changes in the composition of the study cohort due to programming errors that were corrected resulted in different coefficients and confidence intervals for the variables included in the regression. While a few relationships changed, those that did change did not influence or create a need to revise the conclusions of the study. The corrected Table 4 appears below.

**TABLE 4:** Cox proportional hazard regression for time to CMV prophylaxis discontinuation among adults undergoing a first kidney transplant.

Predictors	Overall		High risk (D+/R–)		Intermediate risk (R+)		Low risk (D–/R–)	
	HR (95% CI)	*p*-value	HR (95% CI)	*p*-value	HR (95% CI)	*p*-value	HR (95% CI)	*p*-value
CMV serostatus (vs. D–/R–)								
D+/R–	0.60 (0.56–0.65)	<0.01						
R+	0.96 (0.89–1.03)	0.24						
Time-varying covariates (vs. no condition)								
Neutropenia	1.08 (1.03–1.14)	<0.01	1.06 (0.95–1.18)	0.28	1.09 (1.03–1.16)	<0.01	1.08 (0.88–1.33)	0.47
Leukopenia	1.17 (1.11–1.24)	<0.01	1.22 (1.09–1.37)	<0.01	1.14 (1.07–1.21)	<0.01	1.46 (1.16–1.85)	<0.01
Age 18–64 (vs. age ≥65)	0.84 (0.80–0.87)	<0.01	0.77 (0.70–0.84)	<0.01	0.84 (0.81–0.88)	<0.01	0.86 (0.71–1.03)	0.11
Female (vs. Male)	0.97 (0.93–1.00)	0.05	0.95 (0.87–1.04)	0.25	0.97 (0.93–1.01)	0.11	0.98 (0.84–1.15)	0.83
Race (vs. White)								
African American	1.08 (1.04–1.12)	<0.01	1.02 (0.93–1.12)	0.62	1.07 (1.03–1.12)	<0.01	1.26 (1.06–1.49)	<0.01
Other^a^	0.91 (0.86–0.97)	<0.01	1.17 (0.94–1.46)	0.16	0.89 (0.84–0.95)	<0.01	1.12 (0.77–1.62)	0.56
Region (vs. Northeast)								
Midwest	1.19 (1.13–1.26)	<0.01	1.07 (0.95–1.19)	0.28	1.22 (1.15–1.30)	<0.01	1.12 (0.91–1.39)	0.27
South	1.45 (1.38–1.52)	<0.01	1.16 (1.05–1.29)	<0.01	1.51 (1.43–1.59)	<0.01	1.60 (1.33–1.91)	<0.01
West and Other US territories	1.33 (1.26–1.40)	<0.01	1.15 (1.02–1.30)	0.03	1.38 (1.30–1.47)	<0.01	1.21 (0.95–1.55)	0.12
Primary disease leading to ESRD (vs. diabetes of any type)								
Hypertensive nephrosclerosis	0.99 (0.94–1.05)	0.73	0.96 (0.84–1.10)	0.56	0.99 (0.93–1.05)	0.67	1.10 (0.85–1.43)	0.45
Polycystic kidney disease	1.03 (0.95–1.12)	0.42	0.99 (0.83–1.19)	0.94	1.04 (0.95–1.15)	0.37	1.03 (0.72–1.47)	0.86
Focal glomerular sclerosis	0.99 (0.91–1.07)	0.75	0.90 (0.75–1.09)	0.29	1.04 (0.94–1.14)	0.44	0.76 (0.53–1.09)	0.13
Other	1.00 (0.94–1.06)	0.95	0.98 (0.86–1.12)	0.77	1.00 (0.94–1.07)	0.92	0.98 (0.76–1.26)	0.87
CCI ≥5 (vs. <5)	0.99 (0.94–1.04)	0.58	0.99 (0.88–1.11)	0.85	0.99 (0.94–1.05)	0.76	0.92 (0.75–1.14)	0.44
Comorbid health conditions (vs. absence of condition)								
Cardiovascular disease	1.01 (0.97–1.05)	0.73	0.95 (0.87–1.05)	0.33	1.02 (0.97–1.06)	0.42	1.02 (0.86–1.20)	0.82
Chronic pulmonary disease	1.01 (0.97–1.06)	0.66	1.09 (0.98–1.21)	0.1	0.99 (0.94–1.04)	0.68	1.03 (0.85–1.25)	0.74
Diabetes	0.98 (0.93–1.03)	0.39	1.05 (0.92–1.19)	0.47	0.96 (0.90–1.02)	0.18	1.04 (0.82–1.32)	0.75
Liver disease	0.92 (0.88–0.96)	<0.01	1.00 (0.90–1.12)	0.93	0.90 (0.86–0.95)	<0.01	0.93 (0.76–1.14)	0.49
Rheumatologic disease	1.03 (0.97–1.11)	0.33	1.11 (0.94–1.32)	0.20	1.03 (0.96–1.11)	0.43	0.88 (0.64–1.20)	0.41
Donor type deceased (vs. living)	1.15 (1.09–1.20)	<0.01	1.14 (1.02–1.28)	0.02	1.14 (1.07–1.20)	<0.01	1.20 (0.99–1.45)	0.06
Cold ischemia time <24 h (vs. ≥24 h)	0.97 (0.93–1.01)	0.19	0.93 (0.84–1.03)	0.17	0.97 (0.93–1.02)	0.29	1.01 (0.82–1.24)	0.92
Donor creatinine >1.5 mg/dL (vs. ≤1.5 mg/dL)	0.99 (0.95–1.03)	0.61	0.94 (0.84–1.04)	0.23	1.01 (0.96–1.06)	0.76	0.85 (0.69–1.04)	0.12
Time on dialysis prior to KT in years	0.99 (0.99–1.00)	0.06	1.01 (1.00–1.03)	0.05	0.99 (0.98–1.00)	<0.01	0.99 (0.97–1.02)	0.67
Wait time in years	0.99 (0.98–1.00)	<0.01	0.98 (0.96–1.00)	0.06	0.99 (0.98–1.00)	0.02	1.00 (0.96–1.04)	0.88
PRAs ≥80% (vs. <80%)	0.96 (0.91–1.02)	0.16	1.12 (0.95–1.32)	0.18	0.95 (0.89–1.01)	0.09	0.89 (0.67–1.18)	0.42
HLA A B donor-recipient match ≥3 (vs. <3)	0.97 (0.93–1.01)	0.1	1.00 (0.92–1.10)	0.92	0.95 (0.90–0.99)	0.02	1.10 (0.93–1.30)	0.26
Calendar year of transplant 2011–2013 (vs. 2014–2016)	0.51 (0.46–0.57)							
Induction immunosuppressive therapy^b^ (vs. absence of therapy)								
ATG	0.95 (0.91–1.00)	0.04	0.86 (0.77–0.95)	<0.01	0.96 (0.91–1.01)	0.13	1.15 (0.94–1.40)	0.18
Alemtuzumab	0.95 (0.89–1.00)	0.07	0.92 (0.81–1.06)	0.25	0.96 (0.90–1.03)	0.23	0.88 (0.70–1.12)	0.29
Basiliximab	0.97 (0.92–1.03)	0.31	0.99 (0.88–1.11)	0.82	0.95 (0.90–1.01)	0.12	1.11 (0.89–1.39)	0.36
Other immunosuppression	0.88 (0.74–1.04)	0.13	0.81 (0.48–1.35)	0.41	0.86 (0.72–1.04)	0.12	1.15 (0.63–2.09)	0.66
Maintenance immunosuppressive therapy^c^ (vs. absence of therapy)								
MMF	0.90 (0.82–0.98)	0.01	0.86 (0.72–1.04)	0.13	0.88 (0.79–0.97)	0.01	1.11 (0.78–1.56)	0.56
Tacrolimus	0.80 (0.73–0.87)	<0.01	0.85 (0.71–1.01)	0.07	0.77 (0.70–0.85)	<0.01	0.99 (0.68–1.44)	0.94
AZA, everolimus, and/or cyclosporine	0.89 (0.79–1.01)	0.07	0.86 (0.67–1.10)	0.22	0.88 (0.75–1.02)	0.09	1.37 (0.83–2.26)	0.22
Other immunosuppression	0.98 (0.90–1.06)	0.58	1.04 (0.87–1.24)	0.70	1.00 (0.91–1.10)	0.95	0.79 (0.54–1.16)	0.23
Prednisone or methylprednisolone	0.97 (0.90–1.05)	0.49	1.02 (0.86–1.22)	0.80	1.00 (0.92–1.10)	0.94	0.60 (0.45–0.81)	<0.01

Abbreviations: ATG, antithymocyte globulin; AZA, azathioprine; CCI, Charlson comorbidity index; CI, confidence interval; CMV, cytomegalovirus; D, donor; D+, seropositive donor; D–, seronegative donor; ESRD, end-stage renal disease; HLA, human leukocyte antigen; HR, hazard ratio; KT, kidney transplant; MMF, mycophenolate mofetil; PRA, panel-reactive antibody; R, recipient; R+, seropositive recipient; R–, seronegative recipient; US, United States.

^a^ Other includes Asian, American Indian, Alaska Native, Native Hawaiian, Pacific Islander, multiracial, other, and unknown.

^b^ Other immunosuppression therapies included daclizumab, muromonab-CD3, rituximab, and cyclophosphamide.

^c^ Other immunosuppression maintenance therapies included sirolimus, leflunomide, belatacept, or any other.

In the original article, there was a mistake in **Supplementary Table 1** as published. Changes in the composition of the study cohort due to programming errors that were corrected resulted in different numbers of patients reported throughout the table and slight differences in the proportions of patients in the various subgroups reported. The corrected **Supplementary Table 1** is available at the **Supplementary Material** link of the original paper.

In the original article, there were several errors. Changes in the composition of the study cohort due to programming errors that were corrected resulted in changes to all numeric results. However, conclusions drawn from the corrected analysis have not changed from those originally presented. Below are the changes necessary to correct all paragraphs reporting numeric values from the analysis.

A correction has been made to the **Abstract**:

“Using United States Renal Database System registry data and Medicare claims (1 January 2011–31 December 2017), we examined CMV antiviral use in 20,601 KTRs who received their first KT from 2011 to 2016. Three-quarters of KTRs started CMV prophylaxis (86.9% of high-, 83.6% of intermediate-, and 31.7% of low-risk KTRs). Median time to prophylaxis discontinuation was 121, 90, and 90 days for high-, intermediate-, and low-risk KTRs, respectively. Factors associated with receiving CMV prophylaxis were high-risk status, diabetes, receipt of a well-functioning kidney graft, greater time on dialysis before KT, panel reactive antibodies ≥80%, and use of antithymocyte globulin, alemtuzumab, and tacrolimus. KTRs were more likely to discontinue CMV prophylaxis if they developed leukopenia/neutropenia, had liver disease, or had a deceased donor.”

A correction has been made to **Results**, *Baseline Characteristics*, paragraph 1:

“We identified 67,838 individuals who received their first KT from 2011 to 2016, of whom 20,601 satisfied all inclusion and exclusion criteria (Figure 1). Table 1 summarizes the characteristics of our sample. Most (69.2%) KTRs were at intermediate risk of CMV infection, while 17.0% and 13.8% were at high and low risk, respectively. KTRs were, on average, 53.2 years of age at their initial KT. Most KTRs were male (60.1%) and White (60.0%); one-third were African American. Diabetes (28.4%), hypertensive nephrosclerosis (27.8%), polycystic kidney disease (6.3%), focal glomerular sclerosis (5.6%), and systemic lupus erythematosus (3.6%) were the five most frequent primary diseases leading to ESRD. More than one-third (37.9%) of the KTRs had a CCI score ≥5, and nearly one-quarter of KTRs also had congestive heart failure (23.8%). KTRs spent, on average, 4.8 years on dialysis prior to their KT and 2.6 years on the transplant waiting list. Large proportions of KTRs received their kidney grafts from a deceased donor (81.5%) and were positive for Epstein-Barr virus (82.0%). Most donor kidneys experienced <24 h of cold ischemia time (81.6%) and were well-functioning (donor creatinine clearance ≤1.5 mg/dL). Approximately 22% had HLA A B donor-recipient match scores ≥3, and 9.2% of KTRs had PRA ≥80%. ATG was the most used induction immunosuppressive agent (54.7%), followed by basiliximab (22.2%) and alemtuzumab (16.5%). Almost all KTRs used prednisone and/or methylprednisolone (96.3%), MMF (96.3%), and tacrolimus (94.9%) as maintenance immunosuppressive agents. High-risk KTRs were more likely to have had PRA equal to zero, and high- and intermediate-risk KTRs were less likely to have had three or more HLA A B matches than other KTRs. Intermediate-risk KTRs were slightly older and more likely to be female, African American or Asian, Hispanic, reside in the South or West regions, have diabetes or hypertensive nephrosclerosis as the primary cause of ESRD, have a CCI score ≥5, and PRA ≥80% than KTRs in the other groups. Low-risk KTRs were more likely to reside in the Northeast or Midwest, and they were less likely to have had comorbid diabetes and to have used basiliximab as an induction immunosuppressive agent than other KTRs.”

A correction has been made to **Results**, *Use and Factors Associated with the Use of CMV Antiviral Prophylaxis*, paragraph 1:

“Table 2 displays, and compares across risk groups, the CMV prophylaxis characteristics of KTRs who started CMV prophylaxis. Slightly over three-quarters (77.0%) of KTRs started CMV prophylaxis (86.9% of high-, 83.6% of intermediate-, and 31.7% of low-risk KTRs). Overall, 59.7% and 32.5% of KTRs who started CMV prophylaxis used valganciclovir 450 mg and 900 mg, respectively, while 7.8% used other doses of valganciclovir; no patients used ganciclovir. Overall, KTRs who started prophylaxis did so, on average, 4.2 days after receiving their KTs; time to starting prophylaxis did not vary substantially across risk groups (4.1–4.5 days).”

A correction has been made to **Results**, *Use and Factors Associated with the Use of CMV Antiviral Prophylaxis*, paragraph 2:

“Table 3 displays the results of the logistic regression models for use of CMV prophylaxis (descriptive statistics stratified by CMV prophylaxis status within risk group are available in **Supplementary Table 1**). In general, CMV risk status was the factor most strongly associated with the use of CMV prophylaxis. KTRs who were younger, female, African American or of other races, resided in the Northeast, as well as those whose donor creatinine levels were >1.5 mg/dL, who spent more time on dialysis prior to KT, had PRA ≥80%, and who used ATG, and alemtuzumab were more likely to receive CMV prophylaxis (all and intermediate-risk KTRs). KTRs whose kidney graft experienced cold ischemia time <24 h, used basiliximab, AZA, everolimus, or cyclosporine, or prednisone and/or methylprednisolone were less likely to receive CMV prophylaxis (all and intermediate-risk KTRs). Additionally, high-risk KTRs who had PRA ≥80% were more likely to receive CMV prophylaxis; whereas those with comorbid diabetes, and who used AZA, everolimus, or cyclosporine, MMF or other maintenance immunosuppressive agents were less likely to receive CMV prophylaxis. Low-risk KTRs who were female, resided in the South, and used ATG and alemtuzumab or other immunosuppression as induction immunosuppressive agents were more likely to receive CMV prophylaxis.”

A correction has been made to **Results**, *Duration of Prophylaxis and Factors Associated with Risk of CMV Prophylaxis Discontinuation*, Paragraph 1:

“Figure 2 displays the KM curves for time to prophylaxis discontinuation. The median time to prophylaxis discontinuation (i.e., prophylaxis duration), derived from the KM curves, for the high-risk group of KTRs was longer (121 days) than for intermediate- (90 days) and low-risk (90 days) KTRs. Regardless of type of antiviral agent used, 10.9% of KTRs who used CMV prophylaxis did so for ≥200 days (23.4% and 12.7% of high-risk KTRs who used valganciclovir 450 mg and 900 mg, respectively, did so for ≥200 days) and more than half (55.8%) of high-risk KTRs used CMV prophylaxis for ≥100 days (64.0% and 44.8% of high-risk KTRs who used valganciclovir 450 mg and 900 mg, respectively, did so for ≥100 days). Over one-third (36.5%) of intermediate-risk KTRs used CMV prophylaxis for ≥100 days (39.4% and 23.3% of intermediate-risk KTRs who used valganciclovir 450 mg and 900 mg, respectively, did so for ≥100 days).”

A correction has been made to **Results**, *Duration of Prophylaxis and Factors Associated with Risk of CMV Prophylaxis Discontinuation*, Paragraph 2:

“Table 4 displays the results of the PH Cox regression models for time to CMV prophylaxis discontinuation. We found that, regardless of risk group, KTRs who resided in the South and who developed leukopenia were more likely to discontinue CMV prophylaxis; all KTRs, as well as intermediate-risk group KTRs who developed neutropenia were also more likely to discontinue. Additionally, overall and intermediate-risk KTRs with comorbid liver disease, who experienced a longer wait time, lived in the Midwest, or received MMF or tacrolimus were more likely to discontinue CMV prophylaxis. Among the overall, high-, and intermediate-risk KTRs, those who were younger, received kidney grafts from deceased donors, or lived in the West or other US territories were more likely to discontinue prophylaxis. Finally, overall, intermediate-, and low-risk KTRs who identified as African American were more likely to discontinue CMV prophylaxis, as were overall and intermediate-risk KTRs of other races.”

A correction has been made to **Discussion**, paragraph 1:

“CMV prophylaxis was more common among high- (86.9%) than intermediate- (83.6%) and low-risk (31.7%) KTRs, with all those KTRs using valganciclovir and almost 60% of valganciclovir users using 450 mg per day.”

A correction has been made to **Discussion**, paragraph 2:

“Furthermore, we found that the mean duration of CMV prophylaxis was also longer in our study; however, still only approximately one in five high-risk KTRs completed 200 days of CMV prophylaxis and just over one in three intermediate-risk KTRs completed 100 days of CMV prophylaxis.”

In the original article, there was a mistake in the **Data Availability Statement** as published. The original statement incorrectly stated that the USRDS-Medicare data was publicly available. The corrected Data Availability statement is as follows:

“This study used data from the USRDS-Medicare database, which was provided to the study team subject to the terms of data use agreement (DUA) 2020-41f. The data are not publicly available due to privacy laws and cannot be shared by the authors. However, data obtained from the USRDS-Medicare database for this study may be accessed by applying to USRDS/NIDDK/CMS at usrds@niddk.nih.gov. Upon request, the corresponding author will provide the original data request and the programs used to derive this study’s analytic cohort.”

The authors apologize for these errors and state that this does not change the scientific conclusions of the article. The original article has been updated.

