# Supplementary material for: Real-World Treatment Patterns of Antiviral Prophylaxis for Cytomegalovirus Among Adult Kidney Transplant Recipients: A Linked USRDS-Medicare Database Study
Source: Transpl Int. 2022 Aug 12;35:10528. doi: 10.3389/ti.2022.10528 (PMC9421942; doi:10.3389/ti.2022.10528)
Supplement: Supplementary file 1 [file Table1.docx]

| **Supplemental Table 1.** Characteristics of CMV Prophylaxis among Individuals Undergoing First Kidney Transplant by Serostatus and Prophylaxis Use | | | | | | | | | | | | | |
| --- | --- | --- | --- | --- | --- | --- | --- | --- | --- | --- | --- | --- | --- |
|  | **┌───Overall (N=20,601)───┐** | | | | **┌─High Risk (D+/R–) (N=3,505)─┐** | | | **┌Intermediate Risk (R+) (N=14,256)┐** | | | **┌──Low Risk (D–/R–) (N=2,840)──┐** | | |
| **Characteristic** | **No CMV prophylaxis (N=4,742)** | | **CMV prophylaxis (N=15,859)** | **P-value^a^** | **No CMV prophylaxis (N=459)** | **CMV prophylaxis (N=3,046)** | **P-value^a^** | **No CMV prophylaxis (N=2,343)** | **CMV prophylaxis (N=11,913)** | **P-value^a^** | **No CMV prophylaxis (N=1,940)** | **CMV prophylaxis (N=900)** | **P-value^a^** |
| Mean age in  years (SD) | 54.9  (14.3) | 52.6  (13.9) | | <0.01 | 57.5  (12.6) | 50.8  (14.6) | <0.01 | 56.8  (13.4) | 53.3  (13.5) | <0.01 | 51.9  (15.2) | 50.1  (14.5) | <0.01 |
| Age category in years, N (%) |  |  | |  |  |  |  |  |  |  |  |  |  |
| 18–44 | 1,166 (24.6%) | 4,504 (28.4%) | | <0.01 | 77  (16.8%) | 1,025 (33.7%) | <0.01 | 447  (19.1%) | 3,154 (26.5%) | <0.01 | 642  (33.1%) | 325  (36.1%) | <0.01 |
| 45–64 | 1,974 (41.6%) | 7,571 (47.7%) | |  | 206  (44.9%) | 1,332 (43.7%) |  | 1,018 (43.4%) | 5,844 (49.1%) |  | 750  (38.7%) | 395  (43.9%) |  |
| 65-74 | 1,428 (30.1%) | 3,409 (21.5%) | |  | 162  (35.3%) | 617  (20.3%) |  | 773  (33.0%) | 2,627 (22.1%) |  | 493  (25.4%) | 165  (18.3%) |  |
| ≥75 | 174  (3.7%) | 375  (2.4%) | |  | 14  (3.1%) | 72  (2.4%) |  | 105  (4.5%) | 288  (2.4%) |  | 55  (2.8%) | 15  (1.7%) |  |
| Gender, N (%) |  |  | |  |  |  |  |  |  |  |  |  |  |
| Male | 3,097 (65.3%) | 9,286 (58.6%) | | <0.01 | 329  (71.7%) | 2,138 (70.2%) | 0.52 | 1,407 (60.1%) | 6,544 (54.9%) | <0.01 | 1,361 (70.2%) | 604  (67.1%) | 0.10 |
| Female | 1,645 (34.7%) | 6,573 (41.4%) | |  | 130  (28.3%) | 908  (29.8%) |  | 936  (39.9%) | 5,369 (45.1%) |  | 579  (29.8%) | 296  (32.9%) |  |
| Race, N (%) |  |  | |  |  |  |  |  |  |  |  |  |  |
| White | 3,322 (70.1%) | 9,044 (57.0%) | | <0.01 | 363  (79.1%) | 2,116 (69.5%) | <0.01 | 1,449 (61.8%) | 6,308 (53.0%) | <0.01 | 1,510 (77.8%) | 620  (68.9%) | <0.01 |
| African American | 1,190 (25.1%) | 5,410 (34.1%) | |  | 90  (19.6%) | 842  (27.6%) |  | 709  (30.3%) | 4,320 (36.3%) |  | 391  (20.2%) | 248  (27.6%) |  |
| Asian | 154  (3.2%) | 993  (6.3%) | |  | <11 | 48  (1.6%) |  | 137  (5.8%) | 923  (7.7%) |  | 15  (0.8%) | 22  (2.4%) |  |
| Other^b^ |  |  | |  |  |  |  |  |  |  |  |  |  |
| Hispanic ethnicity, N (%) |  |  | |  |  |  |  |  |  |  |  |  |  |
| Yes | 654 (13.8%) | 3,692 (23.3%) | | <0.01 | 42  (9.2%) | 393  (12.9%) | 0.07 | 483  (20.6%) | 3,159 (26.5%) | <0.01 | 129  (6.6%) | 140  (15.6%) | <0.01 |
| No | 4,048 (85.4%) | 12,045 (76.0%) | |  | 412  (89.8%) | 2,625 (86.2%) |  | 1,838 (78.4%) | 8,666 (72.7%) |  | 1,798 (92.7%) | 754  (83.8%) |  |
| Unknown | 40  (0.8%) | 122  (0.8%) | |  | <11 | 28  (0.9%) |  | 22  (0.9%) | 88  (0.7%) |  | 13  (0.7%) | <11 |  |
| Geographic region, N (%) |  |  | |  |  |  |  |  |  |  |  |  |  |
| Northeast | 823 (17.4%) | 3,007 (19.0%) | | <0.01 | 88  (19.2%) | 632  (20.7%) | <0.01 | 245  (10.5%) | 2,161 (18.1%) | <0.01 | 490  (25.3%) | 214  (23.8%) | <0.01 |
| Midwest | 1,415 (29.8%) | 3,009 (19.0%) | |  | 131  (28.5%) | 684  (22.5%) |  | 679  (29.0%) | 2,143 (18.0%) |  | 605  (31.2%) | 182  (20.2%) |  |
| South | 1,641 (34.6%) | 6,515 (41.1%) | |  | 175  (38.1%) | 1,202 (39.5%) |  | 944  (40.3%) | 4,925 (41.3%) |  | 522  (26.9%) | 388  (43.1%) |  |
| West | 853 (18.0%) | 3,284 (20.7%) | |  | 63  (13.7%) | 526  (17.3%) |  | 473  (20.2%) | 2,650 (22.2%) |  | 317  (16.3%) | 108  (12.0%) |  |
| Other US territories | <11 | 44  (0.3%) | |  | <11 | <11 |  | <11 | 34  (0.3%) |  | <11 | <11 |  |
| Primary diagnosis leading to ESRD,  N (%) |  |  | |  |  |  |  |  |  |  |  |  |  |
| Diabetes mellitus, type 2 | 1,308 (27.6%) | 4,535 (28.6%) | | <0.01 | 138  (30.1%) | 735  (24.1%) | 0.09 | 761  (32.5%) | 3,582 (30.1%) | 0.01 | 409  (21.1%) | 218  (24.2%) | 0.02 |
| Hypertensive nephrosclerosis | 1,215 (25.6%) | 4,509 (28.4%) | |  | 103  (22.4%) | 760  (25.0%) |  | 635  (27.1%) | 3,495 (29.3%) |  | 477  (24.6%) | 254  (28.2%) |  |
| Polycystic kidney disease | 334  (7.0%) | 955  (6.0%) | |  | 35  (7.6%) | 220  (7.2%) |  | 149  (6.4%) | 677  (5.7%) |  | 150  (7.7%) | 58  (6.4%) |  |
| Focal glomerular sclerosis | 259  (5.5%) | 898  (5.7%) | |  | 19  (4.1%) | 202  (6.6%) |  | 119  (5.1%) | 642  (5.4%) |  | 121  (6.2%) | 54  (6.0%) |  |
| Systemic lupus erythematosus | 134  (2.8%) | 617  (3.9%) | |  | <11 | 98  (3.2%) |  | 73  (3.1%) | 488  (4.1%) |  | 51  (2.6%) | 31  (3.4%) |  |
| Diabetes mellitus - Type I | 169  (3.6%) | 551  (3.5%) | |  | 16  (3.5%) | 130  (4.3%) |  | 56  (2.4%) | 371  (3.1%) |  | 97  (5.0%) | 50  (5.6%) |  |
| IGA nephropathy | 175  (3.7%) | 494  (3.1%) | |  | 15  (3.3%) | 110  (3.6%) |  | 75  (3.2%) | 346  (2.9%) |  | 85  (4.4%) | 38  (4.2%) |  |
| Chronic glomerulonephritis unspecified | 113  (2.4%) | 389  (2.5%) | |  | 13  (2.8%) | 75  (2.5%) |  | 54  (2.3%) | 289  (2.4%) |  | 46  (2.4%) | 25  (2.8%) |  |
| Malignant hypertension | 46  (1.0%) | 204  (1.3%) | |  | <11 | 44  (1.4%) |  | 22  (0.9%) | 152  (1.3%) |  | 22  (1.1%) | <11 |  |
| Membranous glomerulonephritis | 53  (1.1%) | 146  (0.9%) | |  | <11 | 40  (1.3%) |  | 26  (1.1%) | 100  (0.8%) |  | 20  (1.0%) | <11 |  |
| Other Disease | 936 (19.7%) | 2,561 (16.1%) | |  | 101  (22.0%) | 632  (20.7%) |  | 373  (15.9%) | 1,771 (14.9%) |  | 462  (23.8%) | 158  (17.6%) |  |
| CCI, N (%) |  |  | |  |  |  |  |  |  |  |  |  |  |
| 0 | 0  (0.0%) | 0  (0.0%) | | 0.26 | 0  (0.0%) | 0  (0.0%) | <0.01 | 0  (0.0%) | 0  (0.0%) | 0.78 | 0  (0.0%) | 0  (0.0%) | 0.21 |
| 1-2 | 1,103 (23.3%) | 3,580 (22.6%) | |  | 85  (18.5%) | 754  (24.8%) |  | 501  (21.4%) | 2,613 (21.9%) |  | 517  (26.6%) | 213  (23.7%) |  |
| 3-4 | 1,889 (39.8%) | 6,221 (39.2%) | |  | 189  (41.2%) | 1,249 (41.0%) |  | 921  (39.3%) | 4,604 (38.6%) |  | 779  (40.2%) | 368  (40.9%) |  |
| ≥5 | 1,750 (36.9%) | 6,058 (38.2%) | |  | 185  (40.3%) | 1,043 (34.2%) |  | 921  (39.3%) | 4,696 (39.4%) |  | 644  (33.2%) | 319  (35.4%) |  |
| Comorbid health conditions, N (%) |  |  | |  |  |  |  |  |  |  |  |  |  |
| Congestive heart failure | 1,135 (23.9%) | 3,777 (23.8%) | | 0.87 | 113  (24.6%) | 669  (22.0%) | 0.2 | 600  (25.6%) | 2,883 (24.2%) | 0.15 | 422  (21.8%) | 225  (25.0%) | 0.06 |
| Diabetes | 1,991 (42.0%) | 7,100 (44.8%) | | <0.01 | 221  (48.1%) | 1,220 (40.1%) | <0.01 | 1,047 (44.7%) | 5,518 (46.3%) | 0.15 | 723  (37.3%) | 362  (40.2%) | 0.13 |
| Diabetes without   chronic   complication | 858 (18.1%) | 3,090 (19.5%) | | 0.03 | 87  (19.0%) | 548  (18.0%) | 0.62 | 422  (18.0%) | 2,380 (20.0%) | 0.03 | 349  (18.0%) | 162  (18.0%) | 0.99 |
| Diabetes with   chronic   complication | 1,868 (39.4%) | 6,718 (42.4%) | | <0.01 | 208  (45.3%) | 1,150 (37.8%) | <0.01 | 987  (42.1%) | 5,233 (43.9%) | 0.11 | 673  (34.7%) | 335  (37.2%) | 0.19 |
| Chronic pulmonary disease | 813 (17.1%) | 2,532 (16.0%) | | 0.05 | 80  (17.4%) | 507  (16.6%) | 0.67 | 416  (17.8%) | 1,872 (15.7%) | 0.01 | 317  (16.3%) | 153  (17.0%) | 0.66 |
| Peripheral vascular disease | 1,123 (23.7%) | 3,902 (24.6%) | | 0.19 | 123  (26.8%) | 726  (23.8%) | 0.17 | 593  (25.3%) | 2,982 (25.0%) | 0.78 | 407  (21.0%) | 194  (21.6%) | 0.73 |
| Rheumatologic disease | 303  (6.4%) | 1,086  (6.8%) | | 0.27 | 25  (5.4%) | 183  (6.0%) | 0.64 | 166  (7.1%) | 850  (7.1%) | 0.93 | 112  (5.8%) | 53  (5.9%) | 0.90 |
| Mild to Moderate liver disease | 644 (13.6%) | 2,372 (15.0%) | | 0.02 | 58  (12.6%) | 424  (13.9%) | 0.46 | 328  (14.0%) | 1,819 (15.3%) | 0.12 | 258  (13.3%) | 129  (14.3%) | 0.45 |
| Severe liver disease | <11 | 82  (0.5%) | | <0.01 | 0  (0.0%) | <11 | 0.62 | <11 | 69  (0.6%) | 0.05 | <11 | <11 | 0.22 |
| Myocardial infarction | 445  (9.4%) | 1,398  (8.8%) | | 0.23 | 55  (12.0%) | 252  (8.3%) | <0.01 | 217  (9.3%) | 1,058  (8.9%) | 0.56 | 173  (8.9%) | 88  (9.8%) | 0.46 |
| Dementia | 43  (0.9%) | 121  (0.8%) | | 0.33 | <11 | 32  (1.1%) | 0.62 | 14  (0.6%) | 82  (0.7%) | 0.62 | 23  (1.2%) | <11 | 0.32 |
| Mean time on dialysis prior to  KT, y (SD) | 4.2  (3.1) | 4.9  (3.2) | | <0.01 | 4.2  (3.1) | 4.7  (3.1) | <0.01 | 4.4  (3.1) | 5.0  (3.3) | <0.01 | 4.0  (3.0) | 4.5  (3.2) | <0.01 |
| Mean wait time in years (SD) | 2.4  (2.0) | 2.6  (2.2) | | <0.01 | 2.4  (2.1) | 2.6  (2.1) | 0.22 | 2.5  (2.1) | 2.6  (2.2) | <0.01 | 2.2  (1.9) | 2.3  (2.0) | 0.16 |
| PRAs, N (%) |  |  | |  |  |  |  |  |  |  |  |  |  |
| 0% | 3,331 (70.2%) | 10,234 (64.5%) | | <0.01 | 337  (73.4%) | 2,161 (70.9%) | 0.08 | 1,609 (68.7%) | 7,457 (62.6%) | <0.01 | 1,385 (71.4%) | 616  (68.4%) | 0.19 |
| 1%–19% | 426  (9.0%) | 1,365  (8.6%) | |  | 47  (10.2%) | 261  (8.6%) |  | 214  (9.1%) | 1,026  (8.6%) |  | 165  (8.5%) | 78  (8.7%) |  |
| 20%–79% | 645 (13.6%) | 2,455 (15.5%) | |  | 57  (12.4%) | 407  (13.4%) |  | 330  (14.1%) | 1,925 (16.2%) |  | 258  (13.3%) | 123  (13.7%) |  |
| 80%–100% | 270  (5.7%) | 1,628 (10.3%) | |  | 16  (3.5%) | 180  (5.9%) |  | 166  (7.1%) | 1,389 (11.7%) |  | 88  (4.5%) | 59  (6.6%) |  |
| Missing | 70  (1.5%) | 177  (1.1%) | |  | <11 | 37  (1.2%) |  | 24  (1.0%) | 116  (1.0%) |  | 44  (2.3%) | 24  (2.7%) |  |
| HLA A B donor-recipient match, N (%) |  |  | |  |  |  |  |  |  |  |  |  |  |
| 0 | 953 (20.1%) | 3,385 (21.3%) | | <0.01 | 102  (22.2%) | 599  (19.7%) | 0.42 | 483  (20.6%) | 2,603 (21.9%) | <0.01 | 368  (19.0%) | 183  (20.3%) | 0.18 |
| 1 | 1,461 (30.8%) | 5,411 (34.1%) | |  | 143  (31.2%) | 1,074 (35.3%) |  | 740  (31.6%) | 4,038 (33.9%) |  | 578  (29.8%) | 299  (33.2%) |  |
| 2 | 1,075 (22.7%) | 3,478 (21.9%) | |  | 109  (23.7%) | 668  (21.9%) |  | 505  (21.6%) | 2,622 (22.0%) |  | 461  (23.8%) | 188  (20.9%) |  |
| 3+ | 1,201 (25.3%) | 3,400 (21.4%) | |  | 99  (21.6%) | 667  (21.9%) |  | 590  (25.2%) | 2,514 (21.1%) |  | 512  (26.4%) | 219  (24.3%) |  |
| Missing | 52  (1.1%) | 185  (1.2%) | |  | <11 | 38  (1.2%) |  | 25  (1.1%) | 136  (1.1%) |  | 21  (1.1%) | 11  (1.2%) |  |
| Hepatitis C seropositivity, N (%) | 145  (3.1%) | 704  (4.4%) | | <0.01 | <11 | 98  (3.2%) | 0.23 | 83  (3.5%) | 565  (4.7%) | 0.01 | 52  (2.7%) | 41  (4.6%) | <0.01 |
| Epstein-Barr virus antibody pos., N (%) | 3,796 (80.1%) | 13,091 (82.5%) | | <0.01 | 347  (75.6%) | 2,390 (78.5%) | 0.17 | 1,845 (78.7%) | 10,019 (84.1%) | <0.01 | 1,604 (82.7%) | 682  (75.8%) | <0.01 |
| Calendar year of transplant, N (%) |  |  | |  |  |  |  |  |  |  |  |  |  |
| 2011 | 475 (10.0%) | 1,382  (8.7%) | | <0.01 | 48  (10.5%) | 290  (9.5%) | 0.07 | 277  (11.8%) | 1,010  (8.5%) | <0.01 | 150  (7.7%) | 82  (9.1%) | 0.03 |
| 2012 | 865 (18.2%) | 2,748 (17.3%) | |  | 90  (19.6%) | 517  (17.0%) |  | 466  (19.9%) | 2,057 (17.3%) |  | 309  (15.9%) | 174  (19.3%) |  |
| 2013 | 818 (17.3%) | 2,734 (17.2%) | |  | 71  (15.5%) | 527  (17.3%) |  | 458  (19.5%) | 2,057 (17.3%) |  | 289  (14.9%) | 150  (16.7%) |  |
| 2014 | 849 (17.9%) | 2,667 (16.8%) | |  | 95  (20.7%) | 514  (16.9%) |  | 430  (18.4%) | 2,011 (16.9%) |  | 324  (16.7%) | 142  (15.8%) |  |
| 2015 | 855 (18.0%) | 3,095 (19.5%) | |  | 81  (17.6%) | 578  (19.0%) |  | 344  (14.7%) | 2,335 (19.6%) |  | 430  (22.2%) | 182  (20.2%) |  |
| 2016 | 880 (18.6%) | 3,233 (20.4%) | |  | 74  (16.1%) | 620  (20.4%) |  | 368  (15.7%) | 2,443 (20.5%) |  | 438  (22.6%) | 170  (18.9%) |  |
| Used immunosuppressive agents, N (%) |  |  | |  |  |  |  |  |  |  |  |  |  |
| Yes | 4,716 (99.5%) | 15,660 (98.7%) | | <0.01 | 453  (98.7%) | 3,013 (98.9%) | 0.67 | 2,327 (99.3%) | 11,765 (98.8%) | 0.02 | 1,936 (99.8%) | 882  (98.0%) | <0.01 |
| No | 26  (0.5%) | 199  (1.3%) | |  | <11 | 33  (1.1%) |  | 16  (0.7%) | 148  (1.2%) |  | <11 | 18  (2.0%) |  |
| Induction therapy,  N (%) |  |  | |  |  |  |  |  |  |  |  |  |  |
| AMG | 2,195 (46.5%) | 8,953 (57.2%) | | <0.01 | 214  (47.2%) | 1,587 (52.7%) | 0.03 | 956  (41.1%) | 6,852 (58.2%) | <0.01 | 1,025 (52.9%) | 514  (58.3%) | <0.01 |
| Basiliximab | 1,422 (30.2%) | 3,096 (19.8%) | | <0.01 | 125  (27.6%) | 680  (22.6%) | 0.02 | 841  (36.1%) | 2,273 (19.3%) | <0.01 | 456  (23.6%) | 143  (16.2%) | <0.01 |
| Alemtuzumab | 674 (14.3%) | 2,695 (17.2%) | | <0.01 | 76  (16.8%) | 524  (17.4%) | 0.75 | 318  (13.7%) | 1,998 (17.0%) | <0.01 | 280  (14.5%) | 173  (19.6%) | <0.01 |
| Rituximab | 19  (0.4%) | 123  (0.8%) | | <0.01 | <11 | <11 | 0.2 | 12  (0.5%) | 105  (0.9%) | 0.07 | <11 | <11 | <0.01 |
| Muromonab-CD3 | <11 | 16  (0.1%) | | 1 | 0  (0.0%) | <11 | 1 | <11 | <11 | 1 | <11 | <11 | 0.38 |
| Daclizumab | <11 | <11 | | 1 | 0  (0.0%) | 0  (0.0%) | NA | <11 | <11 | 0.51 | 0  (0.0%) | 0  (0.0%) | NA |
| Cyclophosphamide |  |  | |  |  |  |  |  |  |  |  |  |  |
| Maintenance therapy, N (%) |  |  | |  |  |  |  |  |  |  |  |  |  |
| Prednisone or methylprednisolone | 4,571 (96.9%) | 15,052 (96.1%) | | 0.01 | 429  (94.7%) | 2,891 (96.0%) | 0.22 | 2,274 (97.7%) | 11,321 (96.2%) | <0.01 | 1,868 (96.5%) | 840  (95.2%) | 0.11 |
| MMF | 4,447 (94.3%) | 15,177 (96.9%) | | <0.01 | 437  (96.5%) | 2,891 (96.0%) | 0.60 | 2,170 (93.3%) | 11,443 (97.3%) | <0.01 | 1,840 (95.0%) | 843  (95.6%) | 0.54 |
| Tacrolimus | 4,350 (92.2%) | 14,977 (95.6%) | | <0.01 | 424  (93.6%) | 2,848 (94.5%) | 0.42 | 2,091 (89.9%) | 11,292 (96.0%) | <0.01 | 1,835 (94.8%) | 837  (94.9%) | 0.90 |
| Cyclosporine | 208  (4.4%) | 191  (1.2%) | | <0.01 | 12  (2.6%) | 58  (1.9%) | 0.31 | 151  (6.5%) | 124  (1.1%) | <0.01 | 45  (2.3%) | <11 | 0.02 |
| Sirolimus | 124  (2.6%) | 115  (0.7%) | | <0.01 | 8  (1.8%) | 37  (1.2%) | 0.35 | 75  (3.2%) | 69  (0.6%) | <0.01 | 41  (2.1%) | <11 | 0.04 |
| Everolimus | 93  (2.0%) | 114  (0.7%) | | <0.01 | 3  (0.7%) | 41  (1.4%) | 0.27 | 61  (2.6%) | 64  (0.5%) | <0.01 | 29  (1.5%) | <11 | 0.31 |
| Belatacept | 86  (1.8%) | 444  (2.8%) | | <0.01 | 15  (3.3%) | 74  (2.5%) | 0.28 | 30  (1.3%) | 351  (3.0%) | <0.01 | 41  (2.1%) | 19  (2.2%) | 0.95 |
| AZA | 18  (0.4%) | 47  (0.3%) | | 0.38 | <11 | <11 | 0.66 | <11 | 33  (0.3%) | 0.39 | <11 | <11 | 0.75 |
| Leflunomide | <11 | <11 | | 0.72 | <11 | <11 | 0.24 | <11 | <11 | 1 | <11 | <11 | 0.53 |
| Other | 63  (1.3%) | 275  (1.8%) | | 0.05 | <11 | 47  (1.6%) | 0.70 | 37  (1.6%) | 211  (1.8%) | 0.50 | 20  (1.0%) | 17  (1.9%) | 0.05 |
| Donor type, N (%) |  |  | |  |  |  |  |  |  |  |  |  |  |
| Deceased | 3,604 (76.0%) | 13,185 (83.1%) | | <0.01 | 382  (83.2%) | 2,525 (82.9%) | 0.86 | 1,871 (79.9%) | 9,995 (83.9%) | <0.01 | 1,351 (69.6%) | 665  (73.9%) | 0.02 |
| Living | 1,138 (24.0%) | 2,674 (16.9%) | |  | 77  (16.8%) | 521  (17.1%) |  | 472  (20.1%) | 1,918 (16.1%) |  | 589  (30.4%) | 235  (26.1%) |  |
| Mean cold ischemia time in hours (SD) | 13.6  (9.7) | 15.3  (10.0) | | <0.01 | 15.2  (10.0) | 14.6  (9.6) | 0.27 | 14.2  (9.6) | 15.6  (10.1) | <0.01 | 12.6  (9.7) | 13.5  (10.2) | 0.02 |
| Cold ischemia time  in hours category, N (%) | |  | |  |  |  |  |  |  |  |  |  |  |
| <24 hours | 3,965 (83.6%) | 12,842 (81.0%) | | <0.01 | 371  (80.8%) | 2,525 (82.9%) | 0.53 | 1,939 (82.8%) | 9,575 (80.4%) | <0.01 | 1,655 (85.3%) | 742  (82.4%) | 0.14 |
| ≥24 hours | 661 (13.9%) | 2,782 (17.5%) | |  | 79  (17.2%) | 472  (15.5%) |  | 355  (15.2%) | 2,182 (18.3%) |  | 227  (11.7%) | 128  (14.2%) |  |
| Missing | 116 (2.4%) | 235 (1.5%) | |  | <11 | 49  (1.6%) |  | 49 (2.1%) | 156  (1.3%) |  | 58  (3.0%) | 30  (3.3%) |  |
| Mean donor creatinine in mg/dL (SD) | 1.0  (0.8) | 1.1  (1.0) | | <0.01 | 1.1  (0.8) | 1.1  (1.1) | 0.28 | 1.0  (0.7) | 1.2  (1.0) | <0.01 | 1.0  (0.8) | 1.2  (1.1) | <0.01 |
| Donor creatinine in  mg/dL category, N (%) | |  | |  |  |  |  |  |  |  |  |  |  |
| ≤1.5 mg/dL | 4,081 (86.1%) | 13,106 (82.6%) | | <0.01 | 389  (84.7%) | 2,546 (83.6%) | 0.23 | 2,010 (85.8%) | 9,807 (82.3%) | <0.01 | 1,682 (86.7%) | 753  (83.7%) | 0.04 |
| >1.5 mg/dL | 654 (13.8%) | 2,745 (17.3%) | |  | 69  (15.0%) | 499  (16.4%) |  | 330  (14.1%) | 2,099 (17.6%) |  | 255  (13.1%) | 147  (16.3%) |  |
| Missing | <11 | <11 | |  | <11 | <11 |  | <11 | <11 |  | <11 | 0 (0.0%) |  |
|  |  |  | |  |  |  |  |  |  |  |  |  |  |
| Mean follow-up duration, months (SD) | 11.8  (1.4) | 11.8  (1.3) | | 0.11 | 11.7  (1.5) | 11.8  (1.2) | 0.28 | 11.7  (1.5) | 11.8  (1.3) | <0.01 | 11.9  (1.1) | 11.8  (1.4) | 0.07 |
| *Abbreviations: ATG = antithymocyte globulin; AZA = azathioprine; CCI = Charlson Comorbidity Index; CMV= cytomegalovirus; D = donor; D+ = seropositive donor;  D– = seronegative donor; ESRD = end-stage renal disease; HLA = human leukocyte antigen; IGA = immunoglobulin A; KT = kidney transplant;  MMF = mycophenolate mofetil; PRA = panel-reactive antibody; R = recipient; R+ = seropositive recipient; R– = seronegative recipient; SD = standard deviation*  *^a^ P-values are compared across patients by type of prophylaxis using t-tests or ANOVA for continuous variables or chi-square tests for categorical variables.*  *^b^ Other includes American Indian, Alaska Native, Native Hawaiian, Pacific Islander, multiracial, other, and unknown* | | | | | | | | | | | | | |
